# Absence seizures and sleep–wake abnormalities in a rat model of *GRIN2B* neurodevelopmental disorder

**DOI:** 10.1111/epi.18606

**Published:** 2025-08-19

**Authors:** Katerina Hristova, Melissa C. M. Fasol, Niamh McLaughlin, Mohammad Sarfaraz Nawaz, Mehmet Taskiran, Ingrid Buller‐Peralta, Anjanette P. Harris, Andrew Sutherland, Alejandro Bassi, Adrian Ocampo‐Garces, Javier Escudero, Peter C. Kind, Alfredo Gonzalez‐Sulser

**Affiliations:** ^1^ Simons Initiative for the Developing Brain, Patrick Wild Centre, Institute for Neuroscience and Cardiovascular Research University of Edinburgh Edinburgh UK; ^2^ Laboratorio de Sueño y Cronobiología, Programa de Fisiologia y Biofisica, Instituto de Ciencias Biomedicas, Facultad de Medicina Universidad de Chile Santiago Chile; ^3^ School of Engineering, Institute for Imaging, Data and Communications University of Edinburgh Edinburgh UK; ^4^ Muir Maxwell Epilepsy Centre University of Edinburgh Edinburgh UK; ^5^ Present address: Moleculent Solna Sweden; ^6^ Present address: ErciyesUniversity Kayseri Turkey

**Keywords:** autism, EEG, epilepsy, NMDAR, SWD

## Abstract

**Objective:**

Pathogenic mutations in *GRIN2B* are an important cause of severe neurodevelopmental disorders resulting in epilepsy, autism, and intellectual disability. *GRIN2B* encodes the GluN2B subunit of *N*‐methyl‐d‐aspartate receptors (NMDARs), which are ionotropic glutamate receptors critical for normal development of the nervous system and synaptic plasticity. Here, we characterized a novel *Grin2b* heterozygous knockout rat model with electroencephalography (EEG) and pharmacological interventions to block spontaneous seizures.

**Methods:**

Through western blot analysis we assessed the extent of GluN2B protein knockdown in knockout (*Grin2b* ^*+/−*^) rats compared to controls. We recorded 24‐h wireless multi‐channel EEG to test whether seizure activity was present and analyzed sleep–wake cycles through a novel automated sleep‐scoring algorithm. We tested the effects of systemic and intracerebral reticular thalamic nucleus administration of ethosuximide, a T‐type voltage‐gated calcium channel blocker, and memantine, a noncompetitive NMDAR antagonist, on seizures.

**Results:**

Compared to wild‐type rats, *Grin2b* ^*+/−*^ rats had a higher incidence of spontaneous spike and wave discharges (SWDs), the electrographic correlate of absence seizures. SWDs were longer in duration and displayed higher delta band spectral power in *Grin2b* ^*+/−*^ animals. Heterozygous animals displayed a reduction in total rapid eye movement sleep and altered distributions of non–rapid eye movement sleep and wake epochs. This was accompanied by a decrease in overall spectral wake power and an increase in beta band power during non–rapid eye movement sleep. The sleep–wake phenotypes were largely uncorrelated with the incidence of SWDs. Systemic ethosuximide reduced the number and duration of SWDs, whereas memantine only reduced their duration. Intrathalamic infusion of both ethosuximide and memantine reduced the number of SWDs.

**Significance:**

Our data show that the new rat *Grin2b* haploinsufficiency model exhibits clinically relevant phenotypes and highlights two potential therapeutic options for *GRIN2B*‐related epilepsy.

**Key points**

1. *Grin2b* heterozygous knockout rats display spontaneous spike and wave discharges, the electrographic correlates of absence seizures.
2. *Grin2b* heterozygous animals have abnormal sleep–wake physiology with specific deficits in rapid eye movement sleep.
3. Systemic treatment with ethosuximide and memantine reduce spike and wave discharge severity in *Grin2b* rats.
4. Intrathalamic treatment with ethosuximide and memantine also blocks spike and wave discharges.

## 1 INTRODUCTION

*GRIN2B* pathogenic variants cause multiple neurodevelopmental clinical phenotypes such as epilepsy, autism spectrum disorder (ASD), intellectual disability, sleep impairments, and movement abnormalities.^1^, ^2^, ^3^, ^4^, ^5^ An estimated 52% of patients with *GRIN2B*‐related neurodevelopmental disorder present with severe epilepsy, which is refractory to therapy in half of diagnosed cases.^1^ A variety of seizure types occur in patients, and include infantile spasms and generalized tonic–clonic, focal, and absence seizures.^1^, ^2^ De novo *GRIN2B* variants are one of the primary monogenic causes of epileptic encephalopathies, in which epilepsy is thought to worsen neurodevelopmental comorbidities.^1^, ^2^, ^6^, ^7^, ^8^ Dysfunctional sleep is common among individuals with *GRIN2B* mutations, and parental reports indicate that at least 60% of patients struggle with initiating and maintaining sleep.^1^, ^5^, ^9^ The predicted incidence of *GRIN2B* pathogenic mutations is 5.91 per 100.000 births,^10^ making it the most prevalent disorder associated with the *N*‐methyl‐d‐aspartate receptor (NMDAR) coding *GRIN* genes.

NMDARs are sodium, potassium, and calcium permeable tetrameric ligand‐gated ion channels, which play a critical role in glutamatergic transmission, synaptic plasticity, and the development of the nervous system.^11^, ^12^ NMDARs are composed of two obligate glycine binding GluN1 subunits and either two GluN2 subunits, which bind to glutamate, or two GluN3 subunits, which bind to glycine.^13^, ^14^ There are four paralogs for GluN2 (GluN2(A–D)), which display differential expression profiles across development and result in varied channel activity dynamics.^11^, ^12^

*GRIN2B* encodes GluN2B, which is highly expressed in prenatal and early postnatal periods, and remains abundant in the forebrain throughout adulthood.^11^, ^12^ Later in development the expression of GluN2A, which is encoded by *GRIN2A*, begins with the two subunits forming di‐ and triheteromeric assemblies at glutamatergic synapses. GluN2B‐containing receptors have a lower open probability, slower deactivation kinetics, and an increased sensitivity to glutamate and glycine compared to GluN2A‐containing receptors.^12^, ^15^ In the context of neurodevelopmental disorders, rodent in vitro data demonstrate that pathogenic *GRIN2B* variants alter NMDAR function,^16^ neuronal migration,^17^ dendrite morphogenesis,^18^, ^19^ and synaptic density,^18^, ^20^ which likely contribute to the aberrant circuit properties that manifest in *GRIN2B*‐related neurodevelopmental disorders.

To test the role of GluN2B on brain network function and the sleep–wake cycle, we performed chronic in vivo wireless electroencephalography (EEG) recordings in a novel rat model of *Grin2b* haploinsufficiency. We identify spontaneously occurring absence seizures, abnormal sleep–wake brain state patterns, and altered spectral properties in mutant animals. We also test the effects of clinically relevant pharmacological treatments on absence seizures.

## 2 METHODS

### 2.1 Animals

All animal procedures were undertaken in accordance with the University of Edinburgh animal welfare committee regulations and were performed under a United Kingdom Home Office project license. Long–Evans *Grin2b* heterozygous knockout rats were generated by the Medical College of Wisconsin gene Editing Rat Resource Center with support from the Simons Foundation Autism Research Initiative (LE‐Grin2bem1Mcwi, RRID:RGD_14394515), and *Grin2b* heterozygous knockouts are hereafter referred to as *Grin2b* ^*+/−*^, whereas wild‐type littermates are *Grin2b* ^*+/+*^. See Supporting Information for additional details.

### 2.2 Surgery

Surface EEG grids and two‐channel EEG head stages were implanted. See Supporting Information for surgical procedures.

### 2.3 EEG recording

All animals were given a minimum of 1‐week post‐surgery recovery before recordings. Rats were habituated to the recording room for at least 24 h. Animals were briefly anesthetized with isoflurane to connect implants to wireless amplifiers, and subsequently recorded for 72 to 96 h (for 24‐h sleep and seizure analysis) or for 4 h (pharmacology experiments) using the TaiNi wireless multichannel recording system (Tainitec, United Kingdom)^21^ at a sampling rate of 250.4 Hz. Experimenters were blind to genotype.

### 2.4 Absence seizure detection and sleep–wake scoring

The electrographic correlate of absence seizures, spike and wave discharges (SWDs) was characterized by periodic high‐amplitude oscillations between 5 and 10 Hz,^22^ which coincided with a spontaneous arrest of animal movement. We first utilized a previously published automatic SWD detector algorithm to quantify SWDs, which uses identification of harmonic peaks at 5–10 Hz in the power spectra to label seizure periods.^23^, ^24^ See Supporting Information for additional details.

### 2.5 Pharmacology experiments

For pharmacology experiments, time‐matched 2‐h or 1‐h periods between 11:00 and 13:00 h across days, were used to analyze the effects of pharmacological treatments on SWDs. Treatment was administered 30 min prior to the start of the recorded and analyzed period (at 10:30 am) and total recording durations for each experimental day did not exceed 4 h. Treatment order was randomized and weighted across animals to minimize the risk of treatment carry‐over effects. See Supporting Information for further details.

### 2.6 Statistical analyses

See Supporting Information for details.

## 3 RESULTS

### 3.1 Gene expression in a novel rat model of *GRIN2B* neurodevelopmental disorder

**FIGURE 1:**
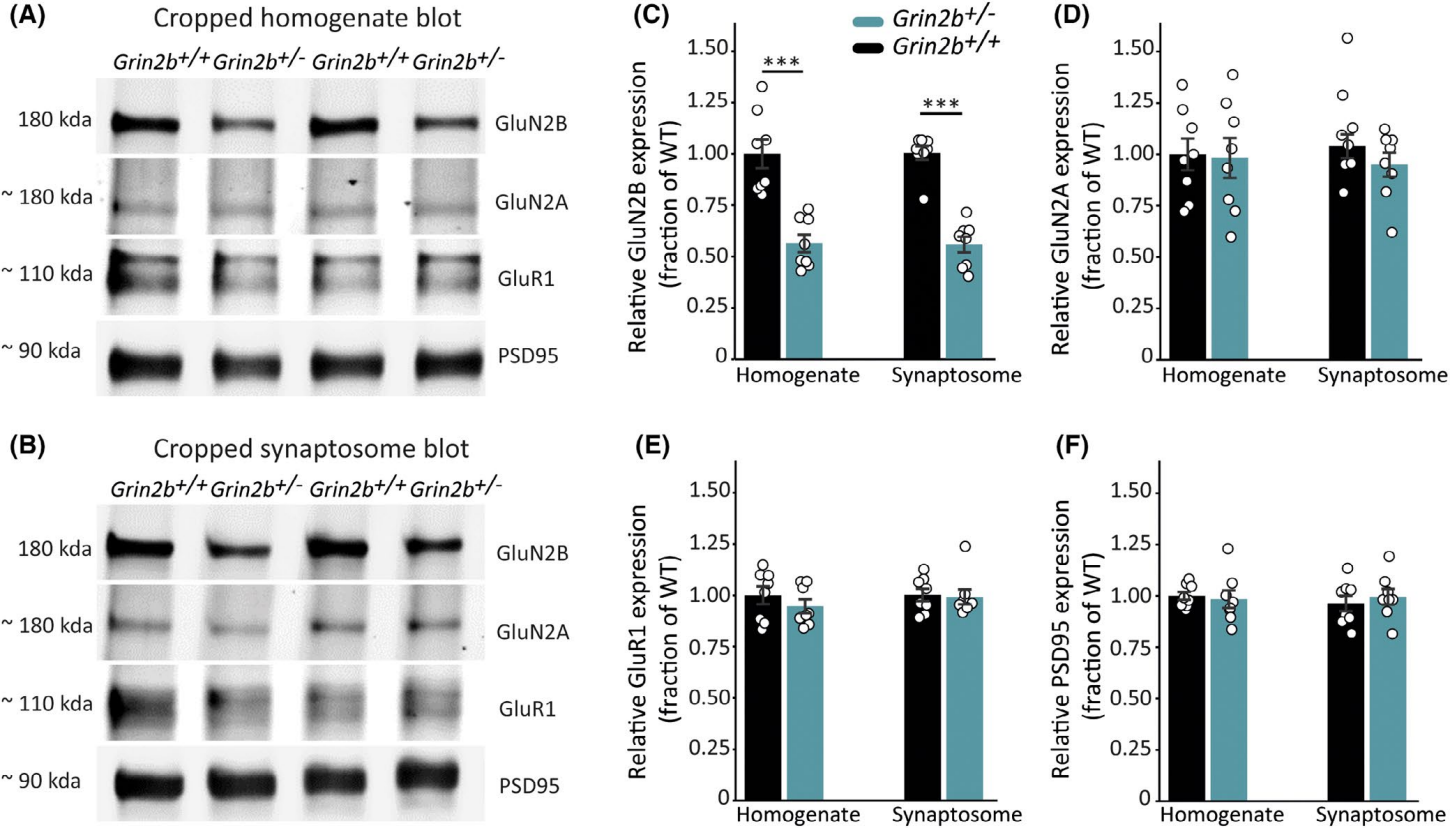
*Grin2b* deletion in rats results in reduction of endogenous GluN2B expression in somatosensory cortex: Representative cropped western blots of extracts from rat somatosensory (A) brain homogenates, and (B) synaptosomes. Full‐length blots are located in Figure S3. Bands in the molecular weight range expected for full‐length GluN2B, GluN2A, GluR1, and PSD95 were detected in homogenates and synaptosomes from wild‐type and *Grin2b* ^*+/−*^ animals. (C) Quantification of GluN2B protein from homogenates and synaptosomes reveals a significant decrease in *Grin2b* ^+/−^ rats (homogenate, two‐sample unpaired *t* test, *df* = 14, *T* = −5.34, *p* = .000104; synaptosome, Wilcoxon rank‐sum test, *W* = 64, *p* = .00094). There was no change in *Grin2b* ^+/−^ rats in expression levels of (D) GluN2A (homogenate, two‐sample unpaired *t* test, *df* = 14, *T* = −.14, *p* = .89; synaptosome, two‐sample unpaired *t* test, *df* = 14, *T* = −1.52, *p* = .15), (E) GluR1 (homogenate, two‐sample unpaired *t* test, *df* = 14, *T* = −.98, *p* = .35; synaptosome, Wilcoxon rank‐sum test, *W* = 28, *p* = .71), or (F) PSD95 (homogenate, two‐sample unpaired *t* test, *df* = 14, *T* = −.35, *p* = .73; synaptosome, two‐sample unpaired *t* test, *df* = 14, *T* = .59, *p* = .56). Bars indicate mean values (mean ± standard error of the mean [SEM]). Points correspond to values from individual rats (*n*_+/+_ = 8, *n*_+/−_ = 8).

We utilized a novel model of *GRIN2B*‐related neurodevelopmental disorder in which the *Grin2b* gene was knocked out of Long–Evans rats. Protein expression was confirmed by immunoblotting somatosensory cortex (Figures 1A and S3A) and hippocampus (Figures S2A and S3C) homogenates, and found to be located at synapses by immunoblotting of somatosensory and hippocampal synaptosome fractions (Figures 1B, S2B, and S3B,D). Glun2B protein levels in homogenates and synaptosome fractions were reduced by ~50% in adult heterozygous knockout (*Grin2b* ^*+/−*^) rats compared to wild‐types (*Grin2b* ^*+/+*^) in somatosensory cortex and hippocampus (Figures 1C and S2C). The expression of GluN2A, GluR1, and PSD95 synaptic proteins in *Grin2b* ^*+/−*^ rats was unaffected in the somatosensory cortex (Figure 1D–F) and the hippocampus (Figure S2D–F).

### 3.2 Normal physical and motor development in *Grin2b* ^*+/−*^ rats

Homozygous *Grin2b* deletion in mice is perinatally lethal, largely due to impaired suckling response.^25^ In contrast, heterozygous *Grin2b* knockout mice are viable and develop normally into adulthood.^25^, ^26^ To determine whether *Grin2b* haploinsufficiency affects postnatal growth and motor development in rats, we assessed physical and behavioral parameters in *Grin2b* ^*+/−*^ rats and wild‐type littermates. We first studied pup survivability by evaluating 16 litters, each consisting of *Grin2b* ^*+/−*^ *and Grin2b* ^*+/+*^ pups. The average survival rate of pups to weaning (prior to genotyping) was 88%. Following weaning, survival rates were determined to be 48% for *Grin2b* ^*+/−*^ rats and 52% for *Grin2b* ^*+/+*^ littermates. Body weight from postnatal Day 3 (P3) to P21 did not differ between genotypes (Figure S4A). Similarly, adult *Grin2b* ^*+/−*^ rats showed normal growth trajectories compared to *Grin2b* ^*+/+*^ controls from 6 to 17 weeks of age (Figure S4B). Pup motor reflex development, measured by righting reflex and negative geotaxis, which test for trunk motor control and vestibular function, respectively,^27^ was not affected by *Grin2b* haploinsufficiency. Righting reflexes, assessed daily from P3 to P7, improved with age in both genotypes and did not differ statistically between *Grin2b* ^*+/−*^ pups and wild‐type littermates (Figure S4C). Furthermore, negative geotaxis responses were comparable between genotypes between P7 and P10 (Figure S4D). Taken together, these findings indicate that *Grin2b* haploinsufficiency does not impair physical development or gross motor function in *Grin2b* ^*+/−*^ rats, which despite having significantly reduced GluN2B expression, appeared fertile and physically indistinguishable from wild‐type littermates.

### 3.3 Absence seizures in *Grin2b* ^*+/−*^ rats

**FIGURE 2:**
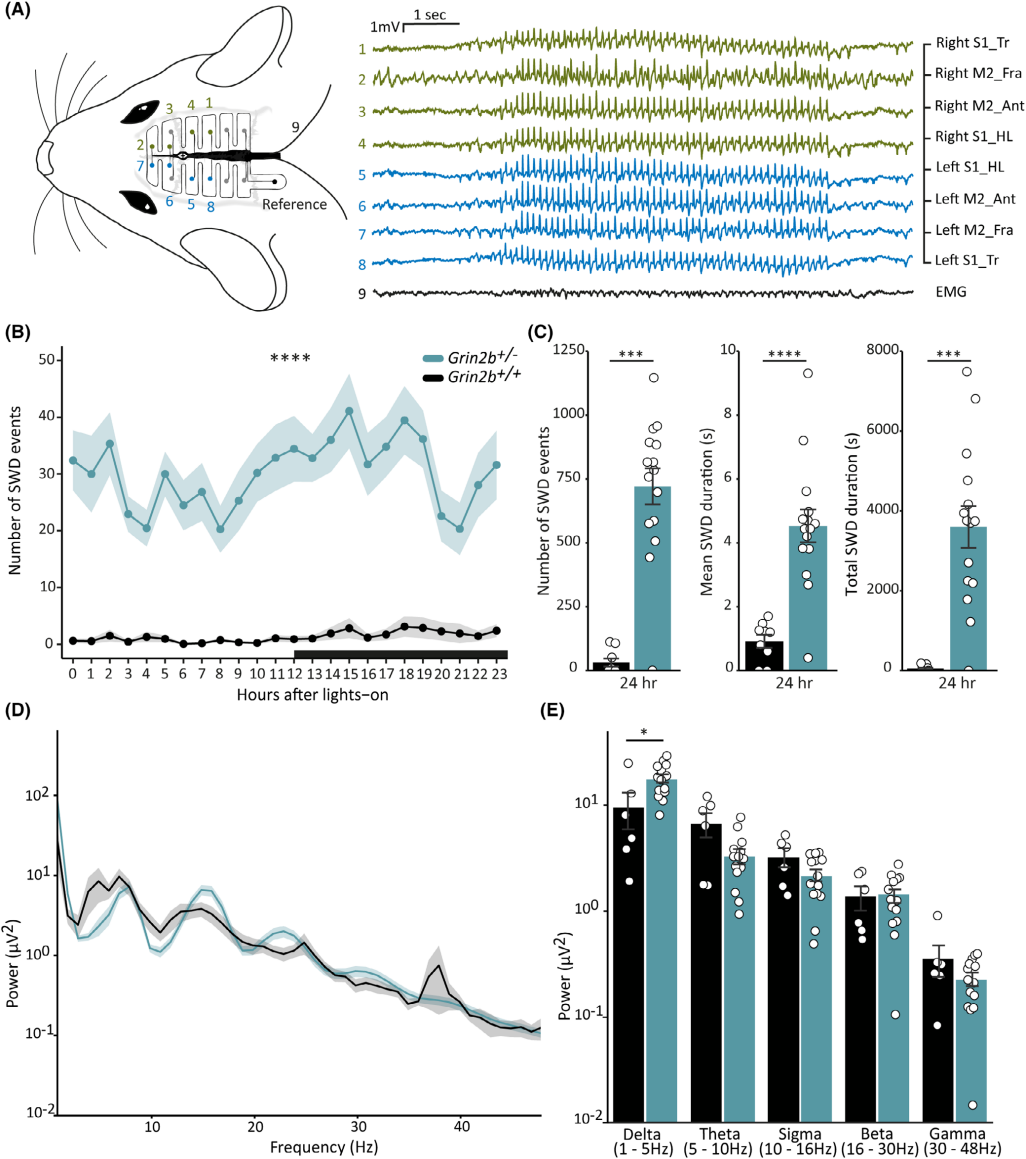
Prevalence and spectral properties of absence seizures in *Grin2b* ^*+/−*^ animals: (A) Schematic of a 16‐channel skull‐surface electroencephalography (EEG) implant illustrating the approximate placement of electrodes relative to the skull (left) and representative EEG and electromyography (EMG) voltage traces from bilateral electrode pairs in the diagram showing a spike and wave discharge (SWD) over the approximate cortical locations (S1‐Tr = Primary somatosensory cortex trunk region, M2‐FrA = Secondary motor cortex frontal association area, M2‐Ant = Secondary motor cortex anterior, S1‐HL = Primary somatosensory cortex hindlimb region) (right). All electrodes are independent of one another and are not interconnected. (B) Average number of SWD events across all rats plotted by hour over the 24‐h light–dark cycle with black bar on *x*‐axis indicating lights off in the animal facility. Hour of the day had no effect on the number of seizures between the two genotypes, and *Grin2b* ^*+/−*^ rats had significantly more seizures overall when compared to wild‐type littermates (linear mixed model, effect of genotype *F* = 55.36, *df* = 1, *p* < .00001; effect of hour *F* = 1.29, *df* = 23, *p* = .16; hour × genotype *F* = .97, *df* = 23 *p* = .49), (**** = *p* ≤ .0001 effect from genotype), (*n*_+/+_ = 9, *n*_+/−_ = 15). Points indicate mean values for all animals (mean ± SEM). (C) Bar plots of total number of SWD events (left), SWD average duration (middle), and total SWD durations (right) average across animals over the entire 24 h, with *Grin2b* ^*+/−*^ rats having significantly higher values (total number of SWDs, Wilcoxon rank‐sum test, *W* = 4.5, *p* = .0002; average SWD duration, two‐sample unpaired *t* test, *df* = 22, *T* = −5.26, *p* = .00003; total SWD durations, Wilcoxon rank‐sum test, *W* = 5.5, *p* = .0002), (**** = *p* ≤ .0001, *** = *p* ≤ .001 effect from genotype), (*n*_+/+_ = 9, *n*_+/−_ = 15). (D) Average EEG power spectra for *Grin2b* ^*+/−*^ rats and wild‐type littermates during all SWD epochs across all animals. Lines represent averages for all animals (mean ± SEM). (E) Quantification of average power in commonly used frequency bands during SWDs, showing an increased delta power in *Grin2b* ^*+/−*^ rats (two‐way analysis of variance [ANOVA], effect of genotype *F* = .53, *df* = 1, *p* = .47; frequency *F* = 72.03, *df* = 4, *p* < .00001; genotype × frequency *F* = 3.27, *df* = 4, *p* = .015; Tukey post hoc test, delta *p* = .039, theta *p* = .34, sigma *p* = .704, beta *p* = .99, gamma *p* = .69), (* = *p* ≤ .05 post hoc test for interaction effect) (*n*_+/+_ = 6, *n*_+/−_ = 14). Bars indicate mean values (mean ± SEM). Points correspond to values from individual rats in C–E.

To determine whether heterozygous knockout of *Grin2b* results in epileptic seizures and if it affects sleep–wake brain states across the circadian cycle, animals were implanted with 14‐channel skull surface electrode grids and electromyography electrodes in the neck muscles (Figure 2A). Wireless freely moving 24‐h recordings were performed in the rats' home cages.

*Grin2b* ^*+/−*^ rats displayed spontaneous behavioral arrests reminiscent of absence seizures in patients. The behavioral arrests occurred simultaneously to electrographically recorded SWDs (Figure 2A and Video S1). Time‐of‐day factors including sleep, circadian rhythm, and light–dark phase dynamics influence seizure expression.^28^, ^29^ We therefore first looked at the distribution of SWDs across each hour of the 24‐h cycle to evaluate whether the occurrence of SWDs in *Grin2b* ^*+/−*^ animals was higher than in wild‐type littermates at specific hours of the day. We then tested whether there were any differences in the number of SWDs during the 12‐h light and dark phases, as collectively the above analyses could inform on relevant seizure activity patterns in *Grin2b* ^*+/−*^ animals. We found that there was a statistically higher number of SWDs in *Grin2b* ^*+/−*^ animals compared to controls across the entire day, and that hour of the day did not impact this difference, indicating that SWD occurrence in both *Grin2b* ^*+/−*^ and wild‐type animals fluctuates in a similar way over the course of the day (Figure 2B). The number of SWD events in *Grin2b* ^*+/−*^ animals did not differ between light and dark phases, and was significantly higher than that observed in wild‐type rats (Figure S5A). The above reflected a statistically significant increase in the total number of SWDs over the full 24 h in *Grin2b* ^*+/−*^ rats when compared to wild‐type *Grin2b* ^*+/+*^ littermates (Figure 2C). Furthermore, *Grin2b* ^*+/−*^ rats had a longer average SWD duration and total SWD duration than *Grin2b* ^*+/+*^ rats (Figure 2C). Notably, we found that SWDs occurred consistently across several generations of *Grin2b* ^*+/−*^ rats (Figure S6A). These results suggest that *Grin2b* haploinsufficiency drives the network to a state of increased absence seizure activity.

We performed spectral analysis on SWDs to determine if spectral features differed between genotypes. In SWD epoch averages we found a clear peak in the theta frequency range with a secondary harmonic in both genotypes (Figure 2D). Spectral power during SWDs in *Grin2b* ^*+/−*^ rats was higher in the delta frequency range when compared to wild‐type littermates (Figure 2E). Filtering for the delta band revealed that the spike component was larger in magnitude in *Grin2b* ^*+/−*^ rats than in wild‐type littermates (Figure S7A,B). The overall increased delta power in *Grin2b* ^*+/−*^ rats may indicate that the mechanisms underlying SWD generation may favor brain states associated with heightened delta activity, such as non–rapid eye movement (NREM) sleep.

To determine whether male and female *Grin2b* ^*+/−*^ rats have different susceptibility to the occurrence of SWDs, we compared both sexes of each genotype from our animal cohort discussed earlier. We found that neither sex nor hour of the day accounted for the increase in SWD events between *Grin2b* ^*+/−*^ and *Grin2b* ^*+/+*^ rats, and that differences were specific to genotype (Figure S8A).

Similarly, although there were still statistically significant differences between genotypes, there were no differences between male and female *Grin2b* ^*+/−*^ rats in the total number of SWDs, average SWD duration, or total time spent in a SWD state (Figure S8B).

### 3.4 Sleep–wake abnormalities in *Grin2b* ^*+/−*^ rats

**FIGURE 3:**
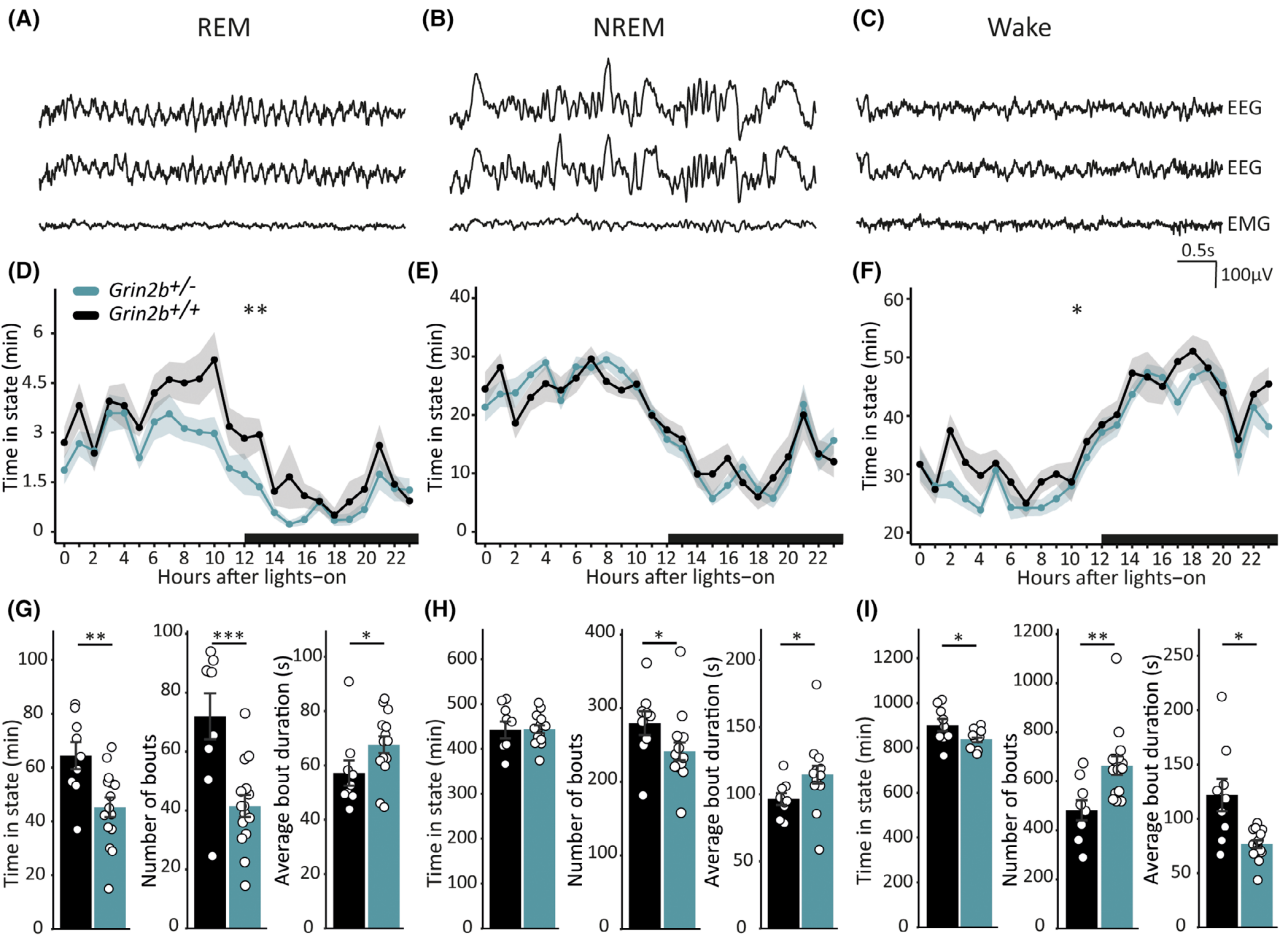
*Grin2b* ^*+/−*^ animals display sleep–wake abnormalities: Representative electroencephalography (EEG) and electromyography (EMG) voltage traces for (A) rapid eye movement (REM), (B) non‐REM (NREM), and (C) wake. Quantification showing the average time spent by hour across all rats in (D) REM, (E) NREM, and (F) wake by hour, across the 24‐h light–dark cycle with black bar on *x*‐axis indicating lights off in the animal facility. *Grin2b* ^*+/−*^ rats had overall significantly less REM sleep compared to wild‐type littermates, and this reduction was not specific to certain hours of the day (linear mixed model, effect of hour *F* = 16.13, *df* = 23, *p* < .00001; genotype *F* = 9.98, *df* = 1, *p* = .0045; hour × genotype *F* = .95, *df* = 23, *p* = .53). No net or individual hour differences were observed between genotypes for NREM (linear mixed model, effect of hour *F* = 21.52, *df* = 23, *p* < .00001; genotype *F* = .0081, *df* = 1, *p* = .93; hour × genotype *F* = .86, *df* = 23, *p* = .66) and wake states (linear mixed model, effect of hour *F* = 22.55, *df* = 23, *p* < .00001; genotype *F* = 7.14, *df* = 1, *p* = .014; hour × genotype *F* = .71, *df* = 23, *p* = .84). ** = effect from genotype. Points indicate mean values for all animals (mean ± standard error of the mean [SEM]) (*n*_+/+_ = 9, *n*_+/−_ = 15). Bar plots of averages across animals showing total time (left), number of bouts (middle), and average bout duration (right) for (G) REM (total minutes, two‐sample unpaired *t* test, *df* = 22, *T* = 3.16, *p* = .0045; number of bouts, two‐sample unpaired *t* test, *df* = 22, *T* = 3.94, *p* = .00069; average bout duration, Wilcoxon rank‐sum test, *W* = 33, *p* = .043), (H) NREM (total minutes, two‐sample unpaired *t* test, *df* = 22, *T* = −.09, *p* = .93; number of bouts, Wilcoxon rank‐sum test, *W* = 29.5, *p* = .025; average bout duration, Wilcoxon rank‐sum test, *W* = 26, *p* = .015), and (I) wake (total minutes, Welch's two‐sample unpaired *t* test, *df* = 10.25, *T* = 2.26, *p* = .047; number of bouts, Wilcoxon rank‐sum test, *W* = 18, *p* = .0035; average bout duration, Welch's two‐sample unpaired *t* test, *df* = 8.91, *T* = 2.97, *p* = .016) during the full 24 h (* = *p* < .05, ** = *p* < .01, *** = *p* < .001). Bars indicate mean values (mean ± SEM). Points correspond to values from individual rats (*n*_+/+_ = 9, *n*_+/−_ = 15).

We quantified minutes spent in REM, NREM, and wake (Figure 3A–C) to determine if *Grin2b* ^*+/−*^ animals exhibited abnormal sleep–wake patterns as reported in patients.^5^, ^9^ Similarly, we tested whether *Grin2b* heterozygous knockout affects the circadian dynamics of brain states. We compared hourly and light–dark phase distributions of sleep–wake states between *Grin2b* ^*+/−*^ and *Grin2b* ^*+/+*^ rats to identify potentially dysregulated sleep–wake rhythms during specific times of the day.

*Grin2b* ^*+/−*^ rats displayed a statistically significant net decrease in REM sleep minutes over the entire 24 h when analyzed hour‐by‐hour relative to wild‐type animals; however, this reduction was not specific to particular hours of the day (Figure 3D). Similarly, *Grin2b* ^*+/−*^ rats spent less total time in REM sleep than wild‐type littermate controls when total minutes per animal were analyzed (Figure 3G). It is likely that this reduction was due to fewer REM sleep bouts in *Grin2b* ^*+/−*^ animals (Figure 3G). Of interest, the average duration of REM sleep bouts was longer for *Grin2b* ^*+/−*^ animals (Figure 3G); however, this was insufficient to compensate for the net REM deficit observed. The significantly reduced REM sleep in *Grin2b* ^*+/−*^ mutants was not specific to light or dark periods (Figure S9A). We did, however, observe significantly fewer REM sleep bouts during both the light and dark periods in *Grin2b* ^*+/−*^ rats, whereas the average duration of REM sleep bouts did not differ between the two phases, between genotypes (Figure S9A).

On an hour‐by‐hour basis the distribution of NREM sleep was not different between *Grin2b* ^*+/−*^ and *Grin2b* ^*+/+*^ rats (Figure 3E). Similarly, there was no difference between *Grin2b* ^*+/−*^ and *Grin2b* ^*+/+*^ rats in the total amount of time spent in NREM sleep over the full 24 h (Figure 3H). Nonetheless, *Grin2b* ^*+/−*^ rats demonstrated abnormal NREM distributions when compared to *Grin2b* ^*+/+*^ littermates, as they had statistically fewer NREM bouts, which where longer in average duration (Figure 3H). During the light and dark phases, however, *Grin2b* ^*+/−*^ animals did not differ from their wild‐type littermates in the total amount of NREM sleep, the amount of NREM bouts, and the average NREM bout duration (Figure S9B).

Finally, we evaluated the distribution of wake across each hour of the light–dark cycle and found that although overall *Grin2b* ^*+/−*^ animals spent significantly reduced time awake, this was not specific to any given hour (Figure 3F). When compared to wild‐type littermates, the total minutes awake was significantly reduced in *Grin2b* ^*+/−*^ rats, with mutants having more wake bouts of shorter duration (Figure 3I). Wake state differences were due to the overall changes across the full 24 h, as the differences between *Grin2b* ^*+/−*^ and *Grin2b* ^*+/+*^ animals occurred irrespective of the light or dark period for the time spent awake, number of wake bouts, and average wake bout duration (Figure S9C).

Sleep states are altered in *Grin2b* ^*+/−*^ rats. Although female animals spent slightly more time in REM sleep and had longer wake bout durations when compared to their male counterparts, these differences were subtle and there were no other statistically significant differences between male and female mutant and wild‐type animals (Table S2 and Figure S10A–F).

**FIGURE 4:**
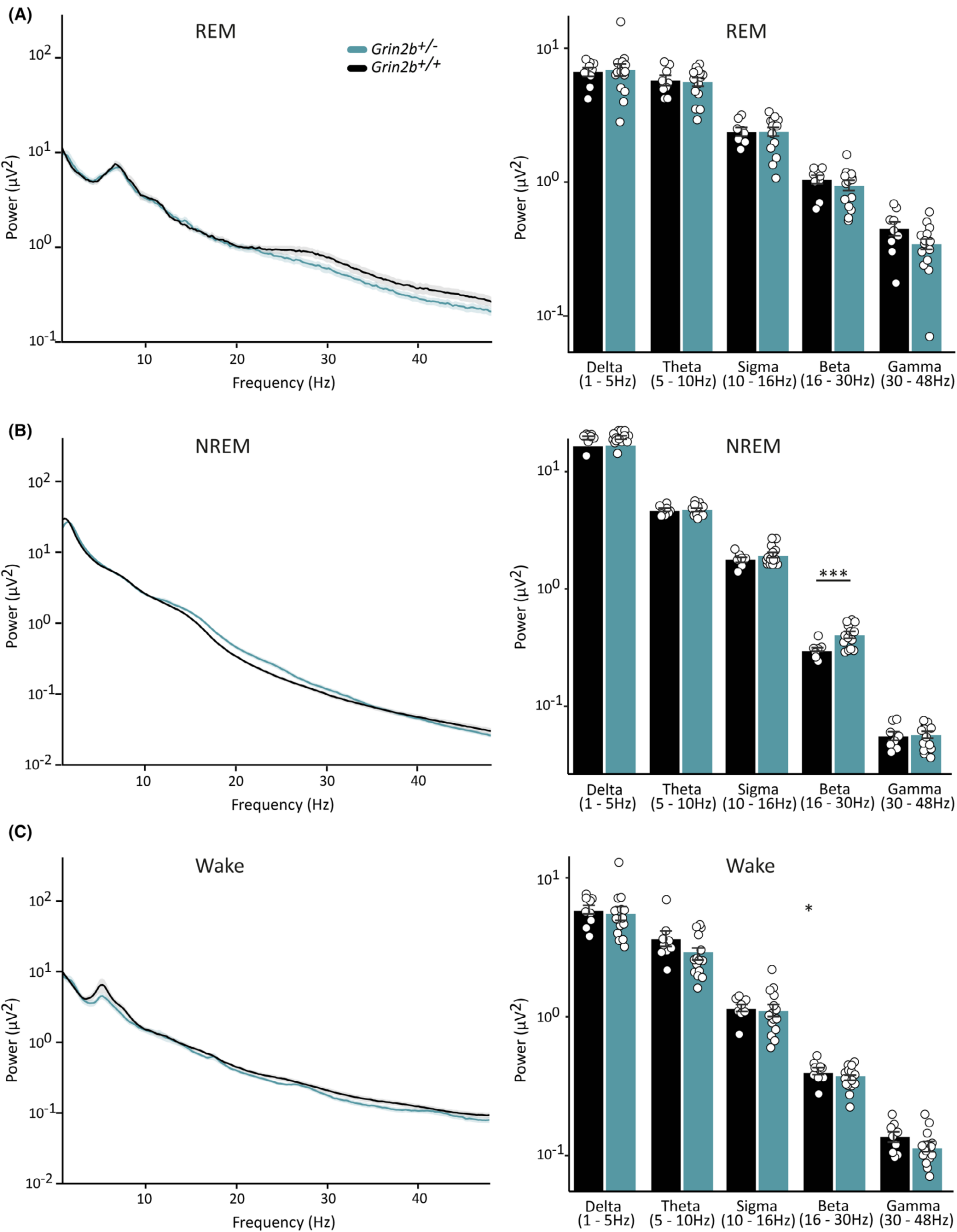
Spectral power is altered in wake and non–rapid eye movement (NREM) sleep in *Grin2b* ^*+/−*^ rats: Average electroencephalography (EEG) power spectra across wild‐type and *Grin2b* ^*+/−*^ rats (left; traces represent averages for all animals (mean ± standard error of the mean [SEM])) and quantification by individual frequency bands (right) during (A) REM, (B) NREM, and (C) wake epochs. Quantification of average power in specific frequency bands during REM epochs showed no spectral differences between *Grin2b* ^+/−^ and wild‐type animals (two‐way analysis of variance [ANOVA], effect of genotype *F* = 2.25, *df* = 1, *p* = .14; frequency *F* = 281.56, *df* = 4, *p* < .00001; genotype × frequency *F* = .57, *df* = 4, *p* = .69). Spectral power in the beta frequency range was significantly higher during NREM epochs in *Grin2b* ^+/−^ rats (two‐way ANOVA, effect of genotype *F* = 5.89, *df* = 1, *p* = .017; frequency *F* = 4037.23, *df* = 4, *p* < .00001; genotype × frequency *F* = 2.64, *df* = 4, *p* = .038; Tukey post hoc test, delta *p* = .98, theta *p* = .99, sigma *p* = .82, beta *p* = .0008, gamma *p* = .99), (*** = post hoc test for interaction). During wake, in *Grin2b* ^+/−^ animals overall power was reduced relative to *Grin2b* ^*+/+*^ littermates, although no significant differences were found in individual frequency bands (two‐way ANOVA, effect of genotype *F* = 6.84, *df* = 1, *p* = .01; frequency *F* = 657.61, *df* = 4, *p* < .00001; genotype × frequency *F* = .33, *df* = 4, *p* = .86), (* = effect from genotype). Bars indicate mean values (mean ± SEM). Points correspond to values from individual rats (*n*_+/+_ = 9, *n*_+/−_ = 14).

We hypothesized that heterozygous *Grin2b* knockout may affect the physiological dynamics of individual brain states. We therefore tested whether spectral properties across REM, NREM, and wake in *Grin2b* ^*+/−*^ rats differed from those in *Grin2b* ^*+/+*^ animals. We calculated the average EEG spectral power from one representative channel across all animals and epochs of each brain state. No statistically significant differences were found between *Grin2b* ^*+/−*^ and *Grin2b* ^*+/+*^ rats during REM sleep (Figure 4A). However, significant differences in spectral power were found for wake and NREM sleep. In NREM sleep, *Grin2b* ^*+/−*^ animals had statistically higher beta power in comparison to wild‐type rats (Figure 4B). In the wake state, spectral power was significantly reduced in *Grin2b* ^*+/−*^ rats relative to *Grin2b* ^*+/+*^ littermates, although this reduction was not restricted to individual frequency bands (Figure 4C). Grouped together, the above results show that *Grin2b* ^*+/−*^ rats have altered sleep–wake brain state distributions and spectral properties.

### 3.5 Brain state abnormalities in *Grin2b* ^*+/−*^ rats are not correlated with absence seizure activity

**FIGURE 5:**
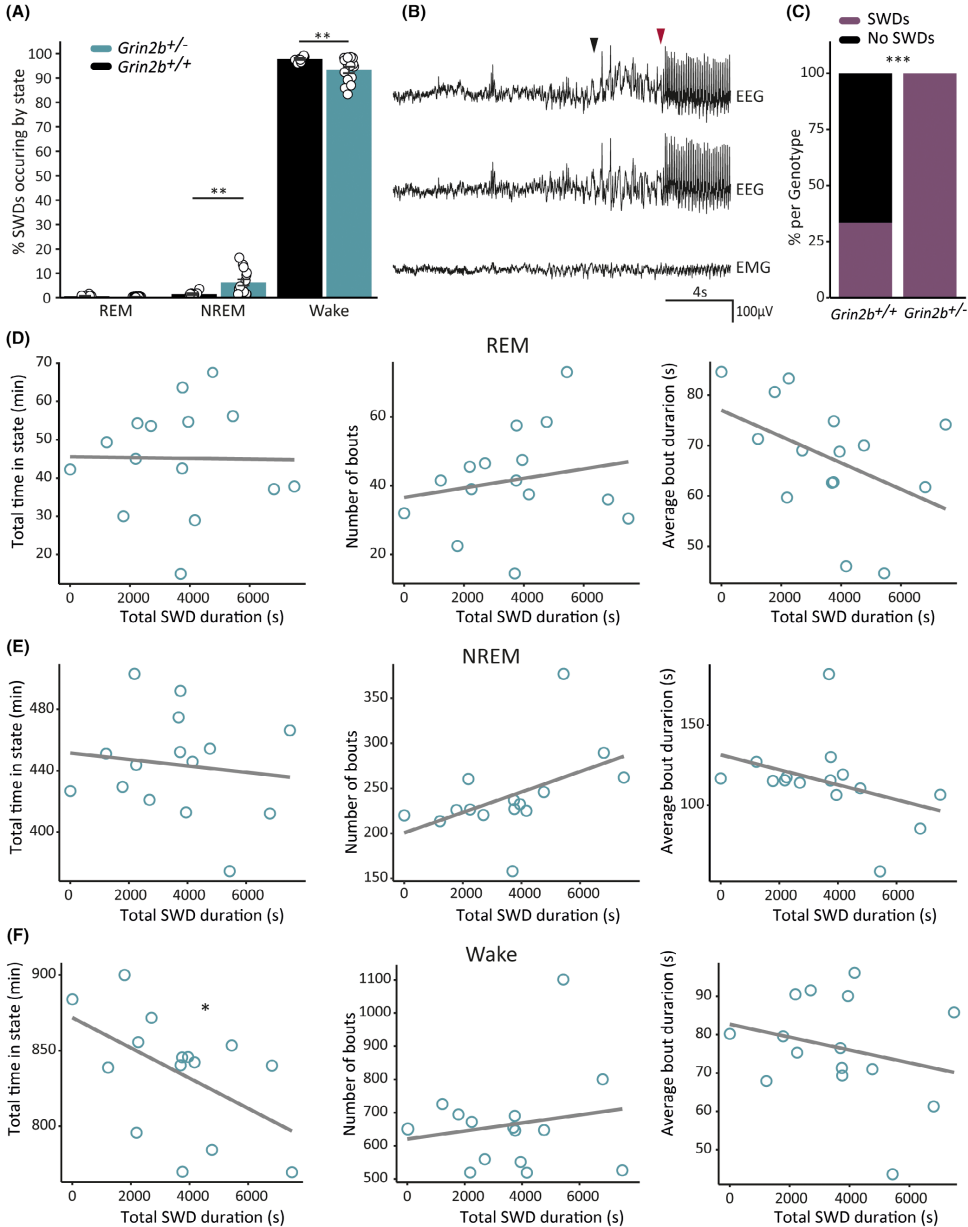
Spike and wave discharges (SWDs) preferentially occur during wake, but sleep abnormalities do not correlate with SWDs in *Grin2b* ^*+/−*^ animals: (A) Plot of average percentage of SWDs occurring during rapid eye movement (REM), non‐REM (NREM), and wake brain states. Compared to wild‐type littermates, *Grin2b* ^*+/−*^ rats have fewer SWDs in wake and more SWDs in NREM (two‐way analysis of variance [ANOVA], effect of genotype *F* = 1, *df* = 1, *p* = .98; state *F* = 5945.056, *df* = 2, *p* < .00001; genotype × state *F* = 10.94, *df* = 2, *p* = .00008; Tukey post hoc test, REM *p* = .82, NREM *p* = .0011, wake *p* = .0022), (**= *p* ≤ .01 post hoc effect from genotype), (*n*_+/+_ = 9, *n*_+/−_ = 15). Bars indicate mean values (mean ± standard error of the mean [SEM]). Points correspond to values from individual rats. (B) Representative electroencephalography (EEG) and electromyography (EMG) voltage traces during a wake‐NREM transition with an SWD. Black arrowhead indicates start of NREM and red arrowhead indicates start of SWD. (C) A higher proportion of SWDs in NREM initiate during wake‐NREM transitions in *Grin2b* ^*+/−*^ when compared to wild‐type animals (Fishers exact test, *p* = .00062), (*** = *p* ≤ .001 effect from genotype), (*n*_+/+_ = 9, *n*_+/−_ = 15). Bars indicate all animals per each genotype and fill color (purple) indicates the relevant proportion of animals with SWDs. Correlation plots of total time in state (left), number of bouts (middle), and average bout duration (right) with SWD duration for (D) REM, (E) NREM, and (F) wake, plotted against total SWD duration. Quantification revealed total SWD duration is negatively correlated with total time awake (Pearson correlation, *T* = −2.16, *df* = 13, *R* = −.51, *p* = .048), (* = *p* ≤ .05), and is uncorrelated to all other sleep measurements: Total time in REM (Pearson correlation, *T* = −.055, *df* = 13, *R* = −.015, *p* = .96) and NREM (Pearson correlation, *T* = −1.47, *df* = 13, *R* = −.13, *p* = .65). Number of REM bouts (Pearson correlation, *T* = .704, *df* = 13, *R* = −.19, *p* = .49), NREM bouts (Pearson correlation, *T* = 2.006, *df* = 13, *R* = .49, *p* = .066), and wake bouts (Pearson correlation, *T* = .61, *df* = 13, *R* = .17, *p* = .55). Average bout duration in REM (Pearson correlation, *T* = .61, *df* = 13, *R* = .17, *p* = .55), NREM (Pearson correlation, *T* = −1.44, *df* = 13, *R* = −.37, *p* = .17), and wake (Pearson correlation, *T* = −.93, *df* = 13, *R* = −.25, *p* = .37). Points correspond to values from individual Grin2b^+/−^ rats (*n*_+/−_ = 15).

We hypothesized that the increased SWD activity in *Grin2b* ^*+/−*^ rats may contribute to the altered sleep–wake brain states. We therefore first tested whether a higher percentage of SWDs preferentially occurred during wake, NREM, or REM sleep and whether there were any differences in this observation between *Grin2b* ^*+/−*^ and *Grin2b* ^*+/+*^ rats. Collectively for both genotypes, we observed that SWDs predominantly occurred during wake (94.8%), followed by NREM (4.4%) and scarcely during REM sleep (.5%) (Figure 5A). Rats have polyphasic sleep patterns, with wake bouts occurring throughout the light and dark phase periods of the 24‐h cycle.^30^ The fact that SWDs occurred predominantly during wake states, and not during specific light‐dark periods (Figure S5A) highlights that SWD occurrence is a more likely brain state than light or dark phase dependent. Of interest, a higher percentage of SWDs are initiated during NREM in *Grin2b* ^*+/−*^ rats than in wild‐type littermates, whereas a higher percentage of seizures in wild‐type animals occurred during the wake state (Figure 5A). The increased frequency of *Grin2b* ^*+/−*^ SWDs in NREM sleep prompted us to examine whether the wake‐NREM transitional state (Figure 5B), when synchronous subcortical output is increased,^31^ is linked to SWD occurrence and if this differed between groups. We tested all SWD epochs identified to originate in NREM and found that all *Grin2b* ^*+/−*^ rats had SWDs initiating at wake‐NREM transitions, whereas this was the case for only a small fraction of *Grin2b* ^*+/+*^ littermates (33.33%, 3/9 rats) (Figure 5C). We subsequently evaluated for differences in the proportion of wake‐NREM transitional SWDs between *Grin2b* ^*+/−*^ animals and wild‐type rats that presented with transitional state seizures. However, we did not observe any variation in the percentage of wake‐NREM transitional SWDs between the two groups: 24.2% of SWDS in *Grin2b* ^*+/−*^ rats occurred during the wake‐NREM transitional period, which was similar to the 22.39% observed in *Grin2b* ^*+/+*^ littermates (Figure S5B).

We hypothesized that there might be a mechanistic link between sleep–wake abnormalities and SWD occurrence. We therefore next determined whether there was a correlation between sleep–wake properties and total SWD duration in *Grin2b* ^*+/−*^ rats. Total seizure duration in *Grin2b* ^*+/−*^ animals did not correlate with total time in REM and NREM sleep (Figure 5D,E). In addition, there was no correlation between total SWD duration and bouts of REM or NREM (Figure 5D,E), as well as average bout duration of REM (Figure 5D) or NREM (Figure 5E). Finally, we found a correlation between *Grin2b* ^*+/−*^ total SWD durations and total time in wake (Figure 5F). This is possibly linked to the statistically decreased time in wake in *Grin2b* ^*+/−*^ rats (Figure 3I), which have much higher numbers of SWDs than controls when awake (Figure 2C). In other words, in *Grin2b* ^*+/−*^animals, SWDs likely replace time in the wake state. There was no correlation, however, between total duration of seizures with wake bouts (Figure 5F) or average wake bout duration (Figure 5F) in *Grin2b* ^*+/−*^ mutants. The general lack of correlation between SWDs and sleep properties in *Grin2b* ^*+/−*^ animals suggests that the two phenotypes may have independent underlying mechanisms.

### 3.6 Pharmacological blockade of SWDs

We next evaluated whether SWDs in *Grin2b* ^*+/−*^ and wild‐type rats could be suppressed pharmacologically. We tested the efficacy of ethosuximide, a T‐type voltage‐gated calcium channel blocker used in the treatment of absence seizures in humans,^32^ which is known to block SWDs in other absence seizure rodent models.^33^ We also evaluated seizure responses to memantine, a noncompetitive NMDAR antagonist currently explored as a mono or adjunctive treatment option in NMDAR‐related epilepsies.^1^, ^34^, ^35^, ^36^ Because *Grin2b* ^*+/−*^ rats had a more severe seizure phenotype, we hypothesized that altered NMDAR‐mediated signaling may contribute to brain circuits underlying SWDs, and that blocking NMDARs would therefore reduce SWD activity.

**FIGURE 6:**
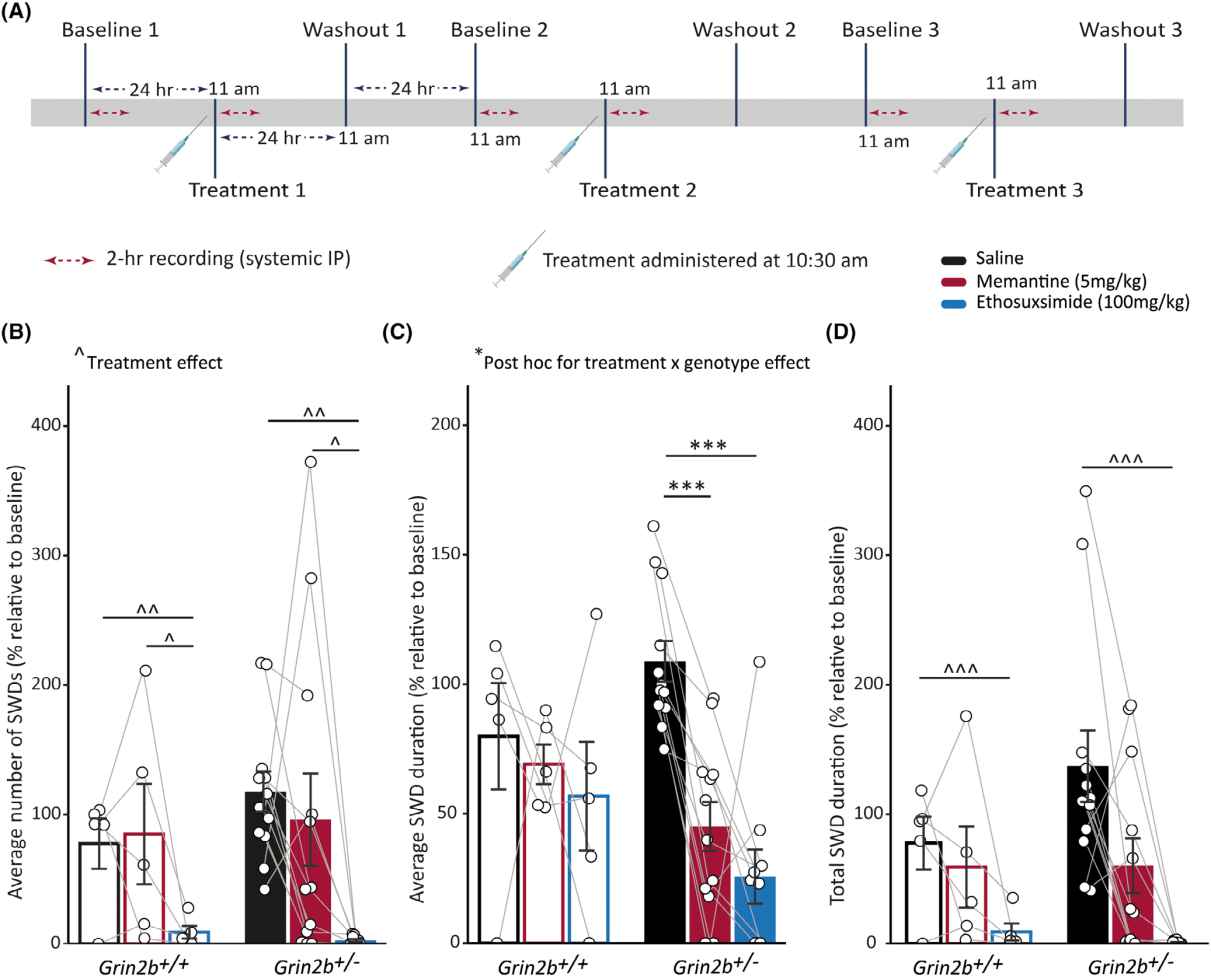
Acute administration of anti‐seizure drugs attenuates spike and wave discharges (SWDs): (A) Diagram of pharmacology experiment timeline. Treatment order is randomized and weighted across animals to minimize the risk of treatment carry‐over effects. Plots showing effects of acute drug treatment on SWD percent relative to baseline, (B) average number, (C) average duration, and (D) total duration in *Grin2b* ^*+/−*^ and wild‐type animals. Treatment with ethosuximide successfully blocked SWD events in both groups (linear mixed model, effect of treatment *F* = 6.48, *df* = 2, *p* = .0049; genotype *F* = .42, *df* = 1, *p* = .53; treatment × genotype *F* = .34, *df* = 2, *p* = .71; Tukey post hoc test for effect of treatment, ethosuximide–saline *p* = .0081, ethosuximide–memantine *p* = .015, and memantine–saline *p* = .96), (^ = *p* ≤ .05, ^^ = *p* ≤ .01 post hoc effect of treatment). Memantine and ethosuximide reduced average SWD duration in Grin2b^+/−^ but not Grin2b^+/+^ rats (linear mixed model, effect of treatment *F* = 9.17, *df* = 2, *p* = .00048; genotype *F* = .71, *df* = 1, *p* = .41; treatment × genotype *F* = 3.34, *df* = 2, *p* = .044; Tukey post hoc test for interaction effect by genotype, *Grin2b* ^*+/−*^ ethosuximide–saline *p* < .0001, Grin2b^+/−^ memantine–saline *p* = .0009, *Grin2b* ^*+/−*^ memantine–ethosuximide *p* = .76, Grin2b^+/+^ ethosuximide–saline *p* = .88, *Grin2b* ^*+/+*^ memantine–saline *p* = .99, and Grin2b^+/+^ memantine–ethosuximide *p* = .99), (* = *p* ≤ .05, *** = *p* ≤ .001 post hoc effect of treatment and genotype interaction). Total seizure duration was also significantly reduced following ethosuximide treatment in both Grin2b^+/−^ and wild‐type rats (linear mixed model, effect of treatment *F* = 9.44, *df* = 2, *p* = .0011; genotype *F* = .67, *df* = 1, *p* = .43; treatment × genotype *F* = 1.15, *df* = 2, *p* = .34; Tukey post hoc test for effect of treatment, ethosuximide‐saline *p* = .0005, ethosuximide–memantine *p* = .083, and memantine–saline *p* = .098), (^^^ = *p* ≤.001 post hoc effect of treatment). Bars indicate mean values (mean ± standard error of the mean [SEM]). Points correspond to values from individual rats and gray lines follow treatment response of each individual rat (*n*_+/+_ = 5, *n*_+/−_ = 12).

We found that in both *Grin2b* ^*+/−*^ and *Grin2b* ^*+/+*^ rats there was a statistically significant reduction in SWDs with pharmacological treatment, as systemic ethosuximide resulted in significantly fewer SWDs than memantine and saline (Figure 6B). Both ethosuximide and memantine, however, reduced the average duration of SWDs in *Grin2b* ^*+/−*^ but not *Grin2b* ^*+/+*^ animals (Figure 6C). The specific effect on *Grin2b* ^*+/−*^ rats, but not wild‐types, may stem from the significantly fewer and shorter seizures observed in *Grin2b* ^*+/+*^ animals. Alternatively, the effect on *Grin2b* ^*+/−*^ rats, but not wild‐types, could be dependent on distinct mechanisms underlying seizures between genotypes. Furthermore, memantine's effect on reducing SWD duration, but not affecting SWD occurrence, may be due to its use‐dependent mechanism of action, requiring opening of the channel to gain access to the channel pore, which happens only after seizures begin.^37^ The combined effect of less SWDs with lower duration with ethosuximide likely resulted in ethosuximide, but not memantine, reducing total seizure durations in both rat groups (Figure 6D). A similar statistically significant reduction in the number and duration of SWDs was observed when absolute, non‐normalized systemic drug effect values were analyzed (Figure S11A–D). Both ethosuximide and memantine could therefore potentially be tested as treatments for SWDs in *GRIN2B* patients.

**FIGURE 7:**
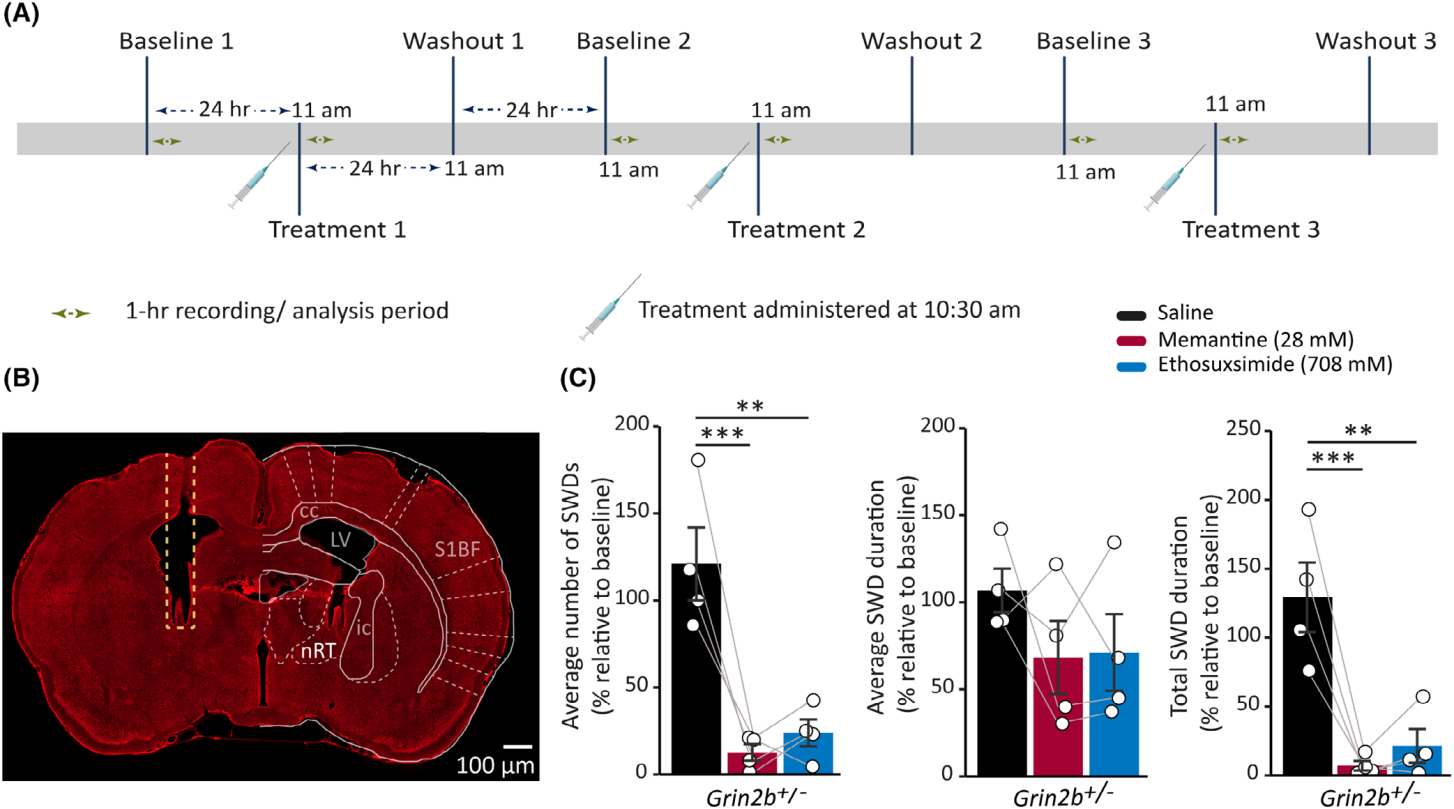
Site‐specific infusion of anti‐seizure drugs in reticular thalamic nucleus (nRT) attenuates spike and wave discharges (SWDs) in *Grin2b* ^*+/−*^ rats: (A) Diagram of pharmacology experiment timeline. Treatments order is randomized and weighted across animals to minimize the risk of treatment carry‐over effects. (B) Representative histology image showing bilaterally implanted cannulas in the rostral nRT (yellow dashed lines), overlaid with approximate coordinates from Paxinos and Watson's Rat Brain Atlas.^41^ (C) Panels showing effects of acute nRT drug infusion on SWD percent relative to baseline average number (left), average duration (middle), and total duration (right) of SWDs in *Grin2b* ^*+/−*^ rats. Treatment with ethosuximide and memantine successfully blocked SWD events in *Grin2b* ^*+/−*^ animals (linear mixed model, effect of treatment *F* = 31.35, *df* = 2, *p* = .00067; Tukey post hoc test, ethosuximide–saline *p* = .0016, ethosuximide–memantine *p* = .75, and memantine–saline *p* = .0009), (** and *** = post hoc tests for treatment effect). Neither drug reduced average SWD durations in *Grin2b* ^*+/−*^ rats (linear mixed model, effect of treatment *F* = 1.41, *df* = 2, *p* = .32). Total seizure duration was also significantly reduced following ethosuximide and memantine infusion to the nRT (linear mixed model, effect of treatment *F* = 31.87, *df* = 2, *p* = .00064; Tukey post hoc test, ethosuximide–saline *p* = .0016, ethosuximide–memantine *p* = .68, memantine–saline *p* = .0008), (** and *** = post hoc tests for treatment effect). Bars indicate mean values (mean ± standard error of the mean [SEM]). Points correspond to values from individual *Grin2b* ^+/−^ rats and gray lines follow treatment response of each individual *Grin2b* ^*+/−*^ rat (*n*_+/−_ = 4). S1BF = Primary somatosensory cortex barrel field, LV = lateral ventricle, cc = corpus callosum, ic = internal capsule.

Previous research demonstrates that inhibiting T‐type calcium channels in the reticular thalamic nucleus (nRT) effectively reduces SWDs in Genetic Absence Epilepsy Rats from Strasbourg (GAERS).^38^, ^39^ In addition, in GAERs, thalamic NMDAR signaling plays a key role in regulating absence seizures of genetic origin, as both NMDAR agonists and antagonists suppress SWD activity.^40^ Therefore, we next investigated the effect of ethosuximide and memantine applied directly to the nRT of *Grin2b* ^*+/−*^ animals. Infusion of ethosuximide and memantine into the nRT of *Grin2b* ^*+/−*^ animals led to a significant decrease in the amount of SWDs compared to saline (Figure 7B,C). Relative to treatment with saline, however, ethosuximide and memantine had no effect on reducing average SWD durations of seizures that did occur (Figure 7B,C). Ultimately, the strong reduction in SWD events following ethosuximide and memantine administration to the nRT, resulted in decreased total seizure durations in *Grin2b* ^*+/−*^ rats, when treated with either drug compared to saline control (Figure 7B,C). Absolute, non‐normalized drug effect values showed a similar trend toward a reduced number and total duration of SWDs in *Grin2b* ^*+/−*^ animals following drug infusion to the nRT (Figure S12A–C). Overall, these data suggest that although both ethosuximide and memantine were able to modulate nRT activity to reduce SWD, likely by inhibiting nRT burst firing,^38^ once seizures are initiated within the thalamocortical circuit, the network dynamics sustaining SWD activity remain largely unaffected.

## 4 DISCUSSION

We identify clinically relevant epileptic and sleep–wake phenotypic abnormalities in a novel model of *GRIN2B* haploinsufficiency. *Grin2b* ^*+/−*^ animals had a 50% reduction in *Grin2b* expression compared to wild‐type levels both in the somatosensory cortex and the hippocampus in adult rats. We show that *Grin2b* ^*+/−*^ rats displayed a higher prevalence of absence seizure SWDs, which had increased delta spectral power, throughout both light and dark phases of the 24‐h cycle. Sleep–wake distributions are disrupted in mutant animals, although the occurrence of SWDs is largely uncorrelated with the severity of sleep–wake abnormalities. Finally, we found that pharmacological treatment with ethosuximide was able to reduce both the number and average duration of absence seizures, whereas memantine only lowers average SWD duration. However, both drugs reduce absence seizure durations only in *Grin2b* ^*+/−*^ animals and not in wild‐types. The difference in seizure susceptibility and drug effects between heterozygous and wild‐type animals could be due to the direct effects of the mutation on seizure‐generating areas or on parallel brain mechanisms. Finally, we demonstrate that the thalamus is critically engaged in the generation of SWDs in *Grin2b* ^*+/−*^ animals by infusing the drugs directly into the nRT.

We show that *Grin2b* ^*+/−*^ rats had a higher number of absence seizures. Over 24 h, *Grin2b* ^*+/−*^ mutants had on average of 59.97 min of SWDs, almost an entire hour of the day in an absence seizure. In contrast, wild‐type littermates had average totals of less than a minute. The Long–Evans rat strain is known to spontaneously exhibit SWDs,^42^, ^43^ which have been described as alpha mu rhythms related to attentiveness and tactile sensory processing.^44^, ^45^ This may explain why, in our study, these events were present in a subset of wild‐type animals. Nevertheless, *Grin2b* haploinsufficiency led to a marked increase in both the number and duration of SWDs compared to wild‐types, suggesting that the mutation further shifts network activity toward a seizure‐prone state, consistent with findings in GAERS rats, where longer SWD durations are associated with greater impairment of consciousness and more severe behavioral disruptions.^46^ Absence seizures have been reported in some individuals with *GRIN2B* missense mutations, although the functional impact of these variants is unknown.^2^

Overall, epilepsy in *GRIN2B*‐related neurodevelopmental disorders has been challenging to model, especially as complete loss of GluN2B‐NMDARs is perinatally lethal. Mice with heterozygous *Grin2b* knockout or a single copy of the *Grin2b*‐C456Y amino‐binding domain variant exhibit behaviors reminiscent of ASD, but notably lack epileptic activity.^4^, ^26^ Adult rats carrying the *Grin2b‐*Trp373 loss‐of‐function variant have a reduced threshold to pentylenetetrazol‐induced seizures; however, they do not present with spontaneous epileptic phenotypes.^47^ Lethal status epilepticus was reported in juvenile mice lacking GluN2B specifically in inhibitory neurons,^48^ and although significant, this observation does not effectively translate to the pan neuronal impact of *GRIN2B* mutations in patients with epilepsy. Hence, the SWDs observed in *Grin2b* ^*+/−*^ mutants here represent the first report of spontaneously occurring seizures in a model with construct validity, highlighting its potential utility for future research into the contribution of heterozygous GluN2B loss to the development and pathophysiology of seizures.

Increased cortical excitation in layer V/VI is thought to trigger SWDs.^22^, ^49^, ^50^, ^51^ Activity then spreads to thalamic structures and generalizes bilaterally through thalamocortical circuits, which compromises reciprocally connected cortex and thalamic nuclei. Within the thalamus, excitatory thalamocortical relay neurons in the ventrobasal region and inhibitory cells in the nRT play crucial roles in modulating circuit oscillations, including SWDs.^52^, ^53^ GluN2B‐containing NMDARs are expressed throughout the thalamocortical circuit^54^, ^55^, ^56^, ^57^, ^58^ and the twofold decrease in GluN2B expression observed in *Grin2b* ^*+/−*^ rats likely alters thalamocortical synaptic dynamics and connectivity, similar to other genetic animal models, in which synaptic transmission within the thalamocortical network is altered and results in SWDs.^59^, ^60^, ^61^, ^62^, ^63^

Clinical reports show a high incidence (>60%) of dysfunctional sleep in individuals with pathological *GRIN2B* variants.^1^, ^5^, ^9^ Nonetheless, quantitative studies that systematically assess sleep in patients with *GRIN2B* mutations are lacking; thus the evidence of dysfunctional sleep in *Grin2b* ^*+/−*^ rats presented here offers valuable insights into the potential sleep impairment phenotypes that may also be found in patient populations. REM sleep was reduced, whereas NREM and wake brain states were abnormal in *Grin2b* ^*+/−*^ rats. The slight reduction in wake minutes is likely due to SWDs occurring primarily during wake, and these times being excluded from wake minute totals.

NMDAR‐dependent signaling plays a critical role in sleep modulation and diurnal rhythmicity. Blocking NMDAR transmission leads to sleep deficits,^64^ whereas activation of these receptors modulates light–dark locomotor and social behavior transitions related to sleep–wake physiology.^65^ Phosphorylation of GluN2B serves as a molecular signature of sleep pressure that modulates sleep cycles.^66^ Moreover, REM sleep is particularly impacted by NMDAR processes, and GluN2B antagonism alters the expression of REM sleep by disrupting gamma oscillations.^67^, ^68^ Given the widespread distribution of GluN2B‐NMDARs across various forebrain structures, the observed REM sleep deficits and altered sleep phenotype in *Grin2b* ^*+/−*^ rats likely result from disruptions to molecular sleep signaling and impairment in NMDAR processes across multiple neural circuits. Furthermore, the lack of correlation between sleep abnormalities and total SWD activity suggests *Grin2b* heterozygous knock‐out may affect thalamocortical seizure and sleep circuits separately.

Mechanistically, absence seizures may result from impaired sleep‐promoting mechanisms, which, along with arousal circuits, regulate thalamocortical excitability.^69^, ^70^ Sleep‐promoting γ‐aminobutyric acid (GABA)ergic activity in the ventrolateral and median preoptic nuclei is reduced in epileptic WAG/Rij rats, whereas median preoptic activation during waking induces SWDs in non‐epileptic rats.^71^ Of note, NMDARs are critical for the function of these sleep‐promoting regions, and regulate NREM and REM sleep expression.^72^, ^73^, ^74^ Absence epilepsy is linked to REM deficits, delayed REM sleep onset, and sleep fragmentation.^75^, ^76^, ^77^ Similar sleep disturbances and SWDs are seen in the WAG/Rij absence epilepsy model^78^, ^79^, ^80^ and in our *Grin2b* ^+/−^ cohort. Furthermore, in *Grin2b* ^+/−^ animals, seizures more often occurred during wake–NREM transitions, suggesting dysfunction in sleep–wake regulatory circuits. Reduced GluN2B expression may therefore compromise sleep‐promoting circuits within the preoptic area. Finally, GluN2B‐containing NMDARs are the dominant postsynaptic receptors facilitating intracortical signaling between layer V pyramidal neurons.^54^ Layer V cells form widespread projections not only to thalamic nuclei but also to other subcortical regions such as the hypothalamus^81^ and brainstem,^82^ which are key sites of vigilance state regulation.^83^ Indeed, it has been suggested that layer V pyramidal neurons integrate sleep‐need signals and coordinate state transitions via their widespread subthalamic projections.^83^ These observations support further investigation into layer V and subcortical sleep–wake circuit dysfunction as likely contributing factors to the sleep and seizure phenotypes in *Grin2b* ^+/−^ rats.

Finally, SWDs in *Grin2b* ^*+/−*^ rats are sensitive to systemic treatment with ethosuximide and memantine. Ethosuximide is one of most widely used drugs for the treatment of absence seizures, and as in this study, successfully diminishes SWDs in rodent models and patients,^39^, ^43^, ^84^, ^85^ mainly through its antagonistic effect on T‐type calcium channels. In contrast, memantine had no effect on the frequency of occurrence of SWDs, but did, however, decrease the average duration of seizures. This effect may be due to memantine's action at the NMDAR magnesium binding site, which is exposed only during periods of membrane depolarization.^36^, ^86^ Consequently, absence seizure initiation may remain unaffected but, after successive bouts of membrane depolarization during an SWD, the events may shorten when memantine blocks the receptor at the magnesium site. Direct infusion of ethosuximide or memantine into the nRT acutely reduced SWD occurrence, likely by shifting thalamic firing output from burst to tonic mode via blockade of T‐type calcium channels and NMDARs. This demonstrates that, as in other rodent models,^38^, ^39^, ^40^, ^61^ the thalamus is critically engaged in SWD generalization and maintenance in *Grin2b* ^*+/−*^ rats.

The effectiveness of memantine on drug‐resistant early‐onset epilepsy in individuals with gain‐of‐function *GRIN2* variants (*GRIN2A, GRIN2B* and *GRIN2D*) is mixed,^1^, ^87^, ^88^, ^89^ and previously, memantine treatment did not improve seizure frequency in four patients with *GRIN2B* gain‐of‐function mutations.^1^ Moreover, memantine is used rarely in patients with loss‐of‐function mutations, although this is consistent with the current understanding of NMDAR pharmacology and the differential effects of gain‐ vs loss‐of‐function mutations on glutamatergic signaling. With the preceding in mind, our data suggest that memantine may be effective for seizure blockade in *Grin2b* haploinsufficiency.

In conclusion, we report on the presence of absence seizures and abnormal sleep architecture in a novel rat model of heterozygous *Grin2b* knockout. In addition, we demonstrate that seizures in *Grin2b* ^*+/−*^ animals are sensitive to ethosuximide and memantine. Overall, the data presented here are consistent with clinical observations and, as such, provide a translationally relevant tool for future research aimed at defining the underlying pathological mechanisms and developing targeted therapeutics for *GRIN2B*‐related disorders.

## AUTHOR CONTRIBUTIONS

A.G.‐S., P.C.K., and J.E. designed the experiments. K.H., M.S.N., A.P.H., A.S., and M.T. performed the experiments. K.H., M.C.M.F., N.M., and M.S.N. analyzed the data. A.G.‐S., M.C.M.F., A.B., and A.O.‐G. developed analysis protocols. K.H. and A.G.‐S. wrote the manuscript with input from all authors.

## CONFLICT OF INTEREST STATEMENT

None of the authors has any conflict of interest to disclose. We confirm that we have read the Journal's position on issues involved in ethical publication and affirm that this report is consistent with those guidelines.

## ETHICS APPROVAL STATEMENT

All animal procedures were undertaken in accordance with the University of Edinburgh animal welfare committee regulations and were performed under a United Kingdom Home Office project license.

## Supporting information


**Table S1.** Comparison of the performance of sleep–wake automated scoring algorithm and visual scoring with results from statistical comparison. Numbers represent epochs scored visually or automatically for rapid eye movement (REM), non‐REM (NREM), and wake states for all animals in the sleep–wake analysis. The diagonal values (bold and gray background) represent instances of agreement between both methods, meaning both produced the same scored state output. Non‐diagonal numbers represent instances of disagreement in state scoring. Total epochs scored, agreement percentage, global agreement across states, and Cohen’s kappa values are shown below scored epoch values.
**Table S2.** Values for statistical analysis across brain states, genotypes and sex. The left‐most column contains description of test statistic and measurements analyzed. Columns 2–4 contain relevant *F*, *df*, and *p*‐values for the effects of genotype and sex, and the interaction effect between them. Values in Rows 3–11 correspond to results from two‐way analysis of variance (ANOVA) test statistics and values in Rows 14–16 correspond to results from Linear mixed models used in hour‐by‐hour time course analysis of sleep–wake distribution. Significant *p*‐values are highlighted in bold text and gray background. Statistical results relate to Figure S7.
**Figure S1.** Percentage agreement between sleep–wake automated scoring algorithm and visual scoring. Agreement between visually and automatically scored epochs of rapid eye movement (REM), non‐REM (NREM), and wake states is 88.1%, 87.5%, and 95%, respectively. Overall agreement between the scoring methods is 90.8%. The kappa coefficient was .83 (±.002 standard error [SE]). See also Table S1 for further details. Bars indicate mean values (mean ± standard error of the mean [SEM]). Points correspond to values from individual rats.
**Figure S2.** Grin2b deletion in rats results in reduction of endogenous *GluN2B* expression in hippocampus. Representative western blots of extracts from rat hippocampal brain (A) homogenates and (B) synaptosomes; full length blots in Figure S3. Bands in the molecular weight range expected for full length GluN2B, GluN2A, GluR1, and PSD95 were detected in homogenates and synaptosomes from wild‐type and Grin2b^+/−^ animals. (C) Quantification of GluN2B protein from homogenates and synaptosomes reveals a significant decrease in Grin2b^+/−^ rats (homogenate, two‐sample unpaired *t* test, *df* = 14, *T* = −897, *p* < .00001; synaptosome, two‐sample unpaired *t* test, *df* = 14, *T* = −3.89, *p* = .0016). There was no change in Grin2b^+/−^ rats in the expression levels of (D) GluN2A (homogenate, two‐sample unpaired *t* test, *df* = 14, *T* = −.16, *p* = .88; synaptosome, two‐sample unpaired *t* test, *df* = 14, *T* = .48, *p* = .64), (E) GluR1 (homogenate, two‐sample unpaired *t* test, *df* = 14, *T* = −1.57, *p* = .14; synaptosome, two‐sample unpaired *t* test, *df* = 14, *T* = −.44, *p* = .67), and (F) PSD95 (homogenate, two‐sample unpaired *t* test, *df* = 14, *T* = .049, *p* = .96; synaptosome, Wilcoxon rank‐sum test, *W* = 28, *p* = .71). Bars indicate mean values (mean ± standard error of the mean [SEM]). Points correspond to values from individual rats (*n*_+/+_ = 8, *n*_+/−_ = 8).
**Figure S3.** Grin2b deletion in rats results in reduction of endogenous *GluN2B* expression in somatosensory cortex and hippocampus. Entire set of western blots of extracts from rat somatosensory brain (A) homogenates and (B) synaptosomes, and extracts from rat hippocampal brain (C) homogenates and (D) synaptosomes. Bands in the molecular weight range expected for full‐length GluN2B, GluN2A, GluR1, and PSD95 were detected in homogenates and synaptosomes from wild‐type and Grin2b^+/−^ animals.
**Figure S4.** No differences in weight and motor development of pup and adult Grin2b^+/−^ and wild‐type rats. (A) Body weight of Grin2b^+/−^ pups from P3 to P21 did not differ from that of Grin2b^+/+^ pup littermates (linear mixed model, effect of genotype *F* = 2.78, *df* = 1, *p* = .12; effect of age *F* = 1769.28, *df* = 3, *p* < .00001; genotype × age *F* = .54, *df* = 3, *p* = .66) (*n*_+/+_ = 5, *n*_+/−_ = 8). (B) Similarly, no genotype‐related differences were observed in adult body weight between 6 and 17 weeks of age (linear mixed model, effect of genotype *F* = .0056, *df* = 1, *p* = .94; effect of age *F* = 715.58, *df* = 11, *p* < .00001; genotype × age *F* = .86, *df* = 11, *p* = .58) (*n*_+/+_ = 47, *n*_+/−_ = 49). Points indicate mean values for all animals (mean ± standard error of the man [SEM]). (C) Grin2b^+/−^ and Grin2b^+/+^ pups exhibited comparable performance in the righting reflex task across developmental days, with righting reflex performance improving significantly over the measured developmental period (linear mixed model, effect of genotype *F* = .29, *df* = 1, *p* = .6; effect of age *F* = 18.36, *df* = 4, *p* < .00001; genotype × age *F* = .57, *df* = 4, *p* = .68) (*n*_+/+_ = 12, *n*_+/−_ = 18). (D) No differences were detected between genotypes on negative geotaxis performance in pups between P7 and P10 (Wilcoxon rank‐sum test, *W* = 87, *p* = .37) (*n*_+/+_ = 12, *n*_+/−_ = 18). Points indicate individual animals. Shaded areas represent the distribution of the data with mean values for each group.
**Figure S5.** The light phase did not influence the prevalence of spike and wave discharges (SWDs) and the percentage of wake/non–rapid eye movement (NREM) transitions did not differ between genotypes. (A) Number of SWD events during the light and dark phases did not differ significantly but were increased significantly in Grin2b^+/−^ rats (linear mixed model, effect of genotype *F* = 55.36, *df* = 1, *p* < .00001; effect of phase *F* = 1.23, *df* = 1, *p* = .28; phase × genotype *F* = .41, *df* = 1, *p* = .53), (*** = effect of genotype). Grin2b^+/−^ rats showed similar SWD event numbers in both the light and dark periods, which significantly exceeded SWD amounts in wild‐type littermates during both phases (effect of genotype *p* < .00001; effect of phase *p* = .28; phase × genotype *p* = .53, linear mixed model) (*n*_+/+_ = 9, *n*_+/−_ = 15). Bars indicate mean values (mean ± standard error of the mean [SEM]). (B) A higher proportion of SWDs in NREM initiate during wake‐NREM transitions in Grin2b^+/−^ animals when compared to wild‐type animals (two‐sample unpaired *t* test, *df* = 16, *T* = −.34, *p* = .74). The amount of SWDs initiating at wake‐NREM transitions was not different between Grin2b^+/−^ rats and Grin2b^+/+^ wild‐types that did have SWDs at transitional periods (3/9 animals) (*p* = .74, two‐sample unpaired *t* test) (*n*_+/+_ = 3, *n*_+/−_ = 15). Bars indicate mean values (mean ± standard error of the mean [SEM]) and points correspond to values from individual rats.
**Figure S6.** Lineage diagram of Grin2b^+/−^ rats. (A) Diagram showing the breeding lineage of Grin2b^+/−^ rats across multiple generations. Teal boxes represent Grin2b colony litters, with Grin2b^+/−^ and Grin2b^+/+^ animals used for breeding or experimental purposes. Teal‐filled boxes indicate litters in which spontaneous spike and wave discharges (SWDs) were detected through electroencephalography (EEG) recording. Teal‐outlined boxes represent Grin2b litters that were not assessed with EEG but were used for breeding. Black‐outlined boxes denote Long–Evans wild‐type outbred litters, whereas purple‐outlined boxes indicate wild‐type litters bred in‐house. This schematic illustrates the persistence of the SWD phenotype across multiple generations.
**Figure S7.** Increased spike and wave discharge (SWD) delta power amplitude in Grin2b^+/−^ rats. (A) Representative electroencephalography (EEG) recording from a Grin2b^+/+^ during an SWD event, showing the raw unfiltered voltage trace (black) alongside the delta bandpass‐filtered trace (green). (B) Corresponding EEG recording from a Grin2b^+/−^ rat during an SWD, with raw unfiltered (black) and delta‐filtered (green) signals. Insets show expanded views of the boxed regions, highlighting SWD morphology and associated delta oscillations, which are of larger amplitude in Grin2b^+/−^ rats (see Figure 2 and Results section).
**Figure S8.** Differences in spike and wave discharge (SWD) properties between male and female Grin2b^+/−^ rats. (A) Number of SWD events plotted by hour over the 24‐h light–dark cycle, with black bar on *x*‐axis indicating lights off in the animal facility. Hour of the day and sex do not impact the amount of SWDs in Grin2b^+/−^ male and female rats, which consistently had significantly more seizures than wild‐type sex‐matched littermates (linear mixed model, effect of hour *F* = 1.24, *df* = 23, *p* = .21; genotype *F* = 47.83, *df* = 1, *p* < .00001; sex *F* = .12, *df* = 1, *p* = .73, hour × genotype × sex *F* = .85, *df* = 23, *p* = .66) (male *n*_+/+_ = 3, female *n*_+/+_ = 6, male *n*_+/−_ = 8, female *n*_+/−_ = 7). Points indicate mean values of all animals (mean ± standard error of the mean [SEM]). (B) Bar plots of total number of SWDs events (left), SWD average duration (middle), and total SWD duration (right). Grin2b^+/−^ rats had a statistically greater number of SWDs, with longer average and total durations than Grin2b^+/+^ rats, and these differences were not influenced by the animals’ sex; SWD number (two‐way analysis of variance [ANOVA], effect of sex *F* = .12, *df* = 1, *p* = .74; genotype *F* = 47.663 *df* = 1, *p* < .0001; sex × genotype *F* = .2, *df* = 1, *p* = .66), SWD average duration (two‐way ANOVA, effect of sex *F* = .0002, *df* = 1, *p* = .99; genotype *F* = 23.17, *df* = 1, *p* = .0001; sex × genotype *F* = .017, *df* = 1, *p* = .89), and total SWD duration (two‐way ANOVA, effect of sex *F* = .0001, *df* = 1, *p* = .99; genotype *F* = 22.9, *df* = 1, *p* = .0001; sex × genotype *F* = .002, *df* = 1, *p* = .96). Bars indicate mean values (mean ± standard error of the mean [SEM]). Points correspond to values from individual rats (male *n*_+/+_ = 3, female *n*_+/+_ = 6, male *n*_+/−_ = 8, female *n*_+/−_ = 7). We do not indicate significance (*) where effect from genotype is found.
**Figure S9.** Reduced rapid eye movement (REM) sleep in Grin2b^+/−^ rats during the light and dark periods. Quantification showing total time (left), number of bouts (middle), and average bout duration (right) for (A) REM, (B) non‐REM (NREM), and (C) wake during the 12‐h light and dark periods. Total time in REM sleep was overall reduced in Grin2b^+/−^ rats compared to wild‐types, and reduced REM sleep was not specific to either light or dark phases (linear mixed model, effect of phase *F* = 119.42, *df* = 1, *p* < .00001; genotype *F* = 9.99, *df* = 1, *p* = .0046; phase × genotype *F* = .85, *df* = 1, *p* = .37). (** = effect of genotype). In comparison to Grin2b^+/+^ animals, the number of REM bouts in Grin2b^+/−^ rats was reduced, both during the light and the dark periods (linear mixed model, effect of phase *F* = 105.93, *df* = 1, *p* < .00001; genotype *F* = 15.52, *df* = 1, *p* = .00067; phase × genotype *F* = 4.53, *df* = 1, *p* = .045; Tukey post hoc test, light *p* = .0001, dark *p* = .032), whereas no differences were found between the two groups in the average length of REM bouts (linear mixed model, effect of phase *F* = 2.26, *df* = 1, *p* = .13; genotype *F* = 1.88, *df* = 1, *p* = .18; phase × genotype *F* = 2.15, *df* = 1, *p* = .16). Analysis by light–dark phase revealed no difference between the two genotypes for total time spent in NREM sleep (linear mixed model, effect of phase *F* = 237.34, *df* = 1, *p* < .00001; genotype *F* = .0065, *df* = 1, *p* = .94; phase × genotype *F* = .94, *df* = 1, *p* = .34), the number of NREM bouts (linear mixed model, effect of phase *F* = 198.28, *df* = 1, *p* < .00001; genotype *F* = 3.62, *df* = 1, *p* = .07; phase × genotype *F* = .041, *df* = 1, *p* = .84), and the average NREM bout durations (linear mixed model, effect of phase *F* = 16.34, *df* = 1, *p* = .00055; genotype *F* = 4.091, *df* = 1, *p* = .055; phase × genotype *F* = .0023, *df* = 1, *p* = .99). Differences between Grin2b^+/−^ rats and littermate controls in total wake minutes were observed, and these differences were not influenced by light or dark phases (linear mixed model, effect of phase *F* = 185.12, *df* = 1, *p* < .00001; genotype *F* = 6.64, *df* = 1, *p* = .013; phase × genotype *F* = .23, *df* = 1, *p* = .64), wake bouts (linear mixed model, effect of phase *F* = 1.39, *df* = 1, *p* = .25; genotype *F* = 9.76, *df* = 1, *p* = .0049; phase × genotype *F* = 1.27, *df* = 1, *p* = .27), or average wake bout duration (linear mixed model, effect of phase *F* = 26.25, *df* = 1, *p* < .00001; genotype *F* = 13.14, *df* = 1, *p* = .0015; phase × genotype *F* = 2.34, *df* = 1, *p* = .14) (* and ** = effect of genotype). Bars indicate mean values (mean ± standard error of the mean [SEM]) and points correspond to values from individual rats (*n*_+/+_ = 9, *n*_+/−_ = 15).
**Figure S10.** Sleep–wake physiology in Grin2b^+/−^ animals does not differ between male and female animals. Quantification showing the time spent in (A) rapid eye movement (REM), (C) non‐REM (NREM), and (E) wake by hour across the 24‐h light–dark cycle; points indicate mean values of all animals (mean ± standard error of the mean [SEM]). Bar plots showing total time (left), number of bouts (middle), and average bout duration (right) for (B) REM, (D) NREM, and (F) wake during the full 24 h; bars indicate mean values (mean ± SEM) and points correspond to values from individual rats. (A) Time in REM is reduced in both female and male Grin2b^+/−^ rats relative to female and male wild‐type littermates, and overall, female rats spent slightly longer in REM sleep than male animals. These differences were not influenced by specific hours of the day (* = effect of sex). (B) For the full 24 h, however, sex did not impact total REM sleep or REM bouts, as each was reduced in both male and female Grin2b^+/−^ mutants relative to their wild‐type sex‐matched littermates. Average REM bout duration was not different between both sexes and genotypes. (C) Time in NREM sleep is similar between Grin2b^+/−^ and wild‐type animals of both sexes throughout each hour of the day. (D) For the 24‐h day, sex did not impact total NREM sleep, NREM bouts, or average NREM bout duration, and these metrics did not differ between male and female Grin2b^+/−^ and Grin2b^+/+^ animals. (E) Time in wake is reduced in both female and male Grin2b^+/−^ rats relative to female and male Grin2b^+/+^ littermates, and this difference is not due to sex or to specific hours of the day. (F) There were no sex‐dependent differences for the total wake time and number of wake bouts when quantified for the full 24 h, whereas average wake bout duration was longer in female rats than in male rats (* = effect of sex). For detailed statistics see Table S2. We do not indicate significance (*) where effect from genotype is found. Bars indicate mean values (mean ± SEM). Points correspond to values from individual rats (male *n*_+/+_ = 3, female *n*_+/+_ = 6, male *n*_+/−_ = 8, female *n*_+/−_ = 7).
**Figure S11.** Acute administration of anti‐seizure drugs attenuates spike and wave discharges (SWDs) in Grin2b^+/−^ and wild‐type animals—absolute value analysis. (A) Diagram of pharmacology experiment timeline. Treatment order is randomized and weighted across animals to minimize the risk of treatment carry‐over effects. Plots showing the effects of acute drug treatment on SWD number (B), average duration (C), and total duration (D) of SWDs in Grin2b^+/−^ and wild‐type animals. Data are presented as absolute values (i.e., not normalized to baseline). Treatment with ethosuximide blocked SWD events in both Grin2b^+/−^ and Grin2b^+/+^ rats (linear mixed model, effect of treatment *F* = 5.69, *df* = 2, *p* = .0091; genotype *F* = 2.08, *df* = 1, *p* = .18; treatment × genotype *F* = .84, *df* = 2, *p* = .44; Tukey post hoc test, ethosuximide–saline *p* = .0074, ethosuximide–memantine *p* = .066, memantine–saline *p* = .61). Memantine and ethosuximide decreased the average duration of SWDs in Grin2b^+/−^ rats, whereas no significant effect was observed in wild‐type controls (linear mixed model, effect of treatment *F* = 15.16, *df* = 2, *p* < .0001; genotype *F* = 2.19, *df* = 1, *p* = .16; treatment × genotype *F* = 5.65, *df* = 2, *p* = .0089; Tukey post hoc test, Grin2b^+/−^ ethosuximide–saline *p* < .0001, Grin2b^+/−^ ethosuximide–memantine *p* = .2, Grin2b^+/−^ memantine–saline *p* < .0001, Grin2b^+/+^ ethosuximide–saline *p* = .5, Grin2b^+/+^ ethosuximide–memantine *p* = .99, Grin2b^+/+^ memantine–saline *p* = .48). Ultimately, ethosuximide led to a significant reduction in total SWD duration in both Grin2b^+/−^ and wild‐type animals (linear mixed model, effect of treatment *F* = 3.85, *df* = 2, *p* = .034; genotype *F* = 2.28, *df* = 1, *p* = .16; treatment × genotype *F* = 1.28, *df* = 2, *p* = .29; Tukey post hoc test, ethosuximide–saline *p* = .025, ethosuximide–memantine *p* = .28, memantine–saline *p* = .45). Bars indicate mean values (mean ± standard error of the mean [SEM]). Points correspond to values from individual rats and gray lines follow treatment response of each individual rat (*n*_+/+_ = 5, *n*_+/−_ = 12).
**Figure S12.** Effects of site‐specific infusion of anti‐seizure drugs in nRT on spike and wave discharges (SWDs) in Grin2b^+/−^ rats—absolute value analysis. (A) Diagram of pharmacology experiment timeline. Treatment order is randomized and weighted across animals to minimize the risk of treatment carry‐over effects (B) Representative histology image showing bilaterally implanted cannulas in the rostral nRT (yellow dashed lines), overlaid with approximate coordinates from Paxinos and Watson’s Rat Brain Atlas (Paxinos & Watson, 2018). (C) Panels showing the effects of acute nRT drug infusion on SWD average number (left), average duration (middle), and total duration (right) of SWDs in Grin2b^+/−^ rats. Treatment with ethosuximide and memantine does not block SWD events in Grin2b^+/−^ animals relative to saline control treatment (linear mixed model, effect of treatment *F* = 3.33, *df* = 2, *p* = .11). Neither drug reduces average SWD durations (linear mixed model, effect of treatment *F* = 1.67, *df* = 2, *p* = .27) or total seizure durations (linear mixed model, effect of treatment *F* = 2.38, *df* = 2, *p* = .17) compared to saline in Grin2b^+/−^ rats. S1BF = primary somatosensory cortex ‐ barrel field, nRT = reticular thalamic nucleus, LV = lateral ventricle, cc = corpus callosum, ic = internal capsule. Bars indicate mean values (mean ± standard error of the mean [SEM]). Points correspond to values from individual rats and gray lines follow treatment response of each individual rat (*n*_+/−_ = 4).

**Video S1.** Example absence seizure in a *Grin2b* ^*+/−*^ rat. Electroencephalography (EEG) recording of spike and wave discharge (SWD) with red bar displaying video synchrony (left). Video recording of rat during absence seizure synchronized in time to red bar (right). Fast head‐bobbing during SWD.

## Data Availability

Raw data are available upon request from the corresponding author. The source code files for the offline sleep state classification and spike and wave discharge (SWD)–detection algorithms are accessible at https://github.com/Gonzalez‐Sulser‐Team/AUTOMATIC‐SLEEP‐SCORER and https://github.com/Gonzalez‐Sulser‐Team/SWD‐Automatic‐Identification, https://zenodo.org/records/12700972.
