# Supplementary material for: Absence seizures and sleep–wake abnormalities in a rat model of GRIN2B neurodevelopmental disorder
Source: Epilepsia. 2025 Aug 19;66(12):4996–5013. doi: 10.1111/epi.18606 (PMC12779324; doi:10.1111/epi.18606)
Supplement: Supplementary file 1 — Table S1. Comparison of the performance of sleep–wake automated scoring algorithm and visual scoring with results from statistical comparison. Numbers represent epochs scored visually or automatically for rapid eye movement (REM), non‐REM (NREM), and wake states for all animals in the sleep–wake analysis. The diagonal values (bold and gray background) represent instances of agreement between both methods, meaning both produced the same scored state output. Non‐diagonal numbers represent instances of disagreement in state scoring. Total epochs scored, agreement percentage, global agreement across states, and Cohen’s kappa values are shown below scored epoch values. Table S2. Values for statistical analysis across brain states, genotypes and sex. The left‐most column contains description of test statistic and measurements analyzed. Columns 2–4 contain relevant F, df, and p‐values for the effects of genotype and sex, and the interaction effect between them. Values in Rows 3–11 correspond to results from two‐way analysis of variance (ANOVA) test statistics and values in Rows 14–16 correspond to results from Linear mixed models used in hour‐by‐hour time course analysis of sleep–wake distribution. Significant p‐values are highlighted in bold text and gray background. Statistical results relate to Figure S7. Figure S1. Percentage agreement between sleep–wake automated scoring algorithm and visual scoring. Agreement between visually and automatically scored epochs of rapid eye movement (REM), non‐REM (NREM), and wake states is 88.1%, 87.5%, and 95%, respectively. Overall agreement between the scoring methods is 90.8%. The kappa coefficient was .83 (±.002 standard error [SE]). See also Table S1 for further details. Bars indicate mean values (mean ± standard error of the mean [SEM]). Points correspond to values from individual rats. Figure S2. Grin2b deletion in rats results in reduction of endogenous GluN2B expression in hippocampus. Representative western blots of extracts [file EPI-66-4996-s001.docx]

**Supplementary Information**

**Absence seizures and sleep abnormalities in a rat model of *GRIN2B* neurodevelopmental disorder**

Katerina Hristova^1,†^, Melissa Fasol^1,†^, Niamh McLaughlin^1^, Mohammad Sarfaraz Nawaz^1^, Mehmet Taskiran^1^, Ingrid Buller-Peralta^1^, Anjanette P. Harris^1^, Andrew Sutherland^1^, Alejandro Bassi^2^, Adrian Ocampo-Garces^2^, Javier Escudero^3^, Peter C. Kind^1^, Alfredo Gonzalez-Sulser^1^

^†^These authors contributed equally to the work.

**Content**

**Supplementary Table 1**

**Supplementary Table 2**

**Supplementary Figure 1**

**Supplementary Figure 2**

**Supplementary Figure 3**

**Supplementary Figure 4**

**Supplementary Figure 5**

**Supplementary Figure 6**

**Supplementary Figure 7**

**Supplementary Figure 8**

**Supplementary Figure 9**

**Supplementary Figure 10**

**Supplementary Figure 11**

**Supplementary Figure 12**

**Supplementary Methods**

|  | ***Visually scored epochs*** | | |  |  |
| --- | --- | --- | --- | --- | --- |
|  | *REM* | *NREM* | *Wake* | |  |
| **Automatically Scored Epochs** |  |  |  | |  |
| *REM* | **2378** | 107 | 233 | |  |
| *NREM* | 119 | **20637** | 781 | |  |
| *Wake* | 201 | 285 | **19254** | |  |
| *Total* | 2698 | 23594 | 20268 | |  |
| *Agreement (%)* | 88.1 | 87.5 | 95 | |  |
| *Global agreement (%)* | 90.8 | |  |  |  |
| *Cohen's kappa (κ) (± Se_κ_)* | 0.83 ± 0.002 | |  |  |  |

***Supplementary Table 1 Comparison of the performance of sleep-wake automated scoring algorithm and visual scoring with results from statistical comparison.*** *Numbers represent epochs scored visually or automatically for REM, NREM and wake states for all animals in sleep-wake analysis. The diagonal values (bold and grey background) represent instances of agreement between both methods, meaning both produced the same scored state output. None-diagonal numbers represent instances of disagreement in state scoring. Total epochs scored, agreement percentage, global agreement across states and Cohen’s kappa values are shown below scored epoch values.*

| Two-way ANOVA | *Effect of genotype* | | | *Effect of sex* | | | *Effect of genotype x sex* | | |
| --- | --- | --- | --- | --- | --- | --- | --- | --- | --- |
|  | *F* | *DF* | *P* | *F* | *DF* | *P* | *F* | *DF* | *P* |
| *Total REM duration* | 8.23 | 1 | **0.0095** | 0.0094 | 1 | 0.92 | 0.83 | 1 | 0.37 |
| *Number of REM bouts* | 12.46 | 1 | **0.0021** | 0.033 | 1 | 0.86 | 0.39 | 1 | 0.54 |
| *Average REM duration* | 2.49 | 1 | 0.13 | 1.76 | 1 | 0.41 | 0.036 | 1 | 0.85 |
| *Total NREM duration* | 0.0074 | 1 | 0.93 | 0.46 | 1 | 0.5 | 0.064 | 1 | 0.8 |
| *Number of NREM bouts* | 2.96 | 1 | 0.101 | 0.0066 | 1 | 0.94 | 0.0045 | 1 | 0.95 |
| *Average NREM duration* | 0.28 | 1 | 0.11 | 0.83 | 1 | 0.37 | 0.0025 | 1 | 0.96 |
| *Total wake duration* | 5.054 | 1 | **0.036** | 1.66 | 1 | 0.21 | 0.12 | 1 | 0.73 |
| *Number of wake bouts* | 7.13 | 1 | **0.015** | 3.77 | 1 | 0.066 | 0.025 | 1 | 0.62 |
| *Average wake duration* | 10.38 | 1 | **0.0043** | 5.69 | 1 | **0.027** | 1.9 | 1 | 0.18 |
| *Linear mixed model* |  | | |  | | | ***Effect of genotype x sex x hour*** | | |
|  |  |  |  |  |  |  | *F* | *DF* | *P* |
| *REM hour-by-hour* | 8.84 | 1 | **0.0075** | 0.85 | 1 | **0.037** | 1.24 | 1 | 0.21 |
| *NREM hour-by-hour* | 0.043 | 1 | 0.52 | 0.45 | 1 | 0.5 | 1.46 | 1 | 0.078 |
| *Wake hour-by-hour* | 5.16 | 1 | **0.038** | 1.76 | 1 | 0.41 | 1.31 | 1 | 0.16 |

***Supplementary Table 2 Values for statistical analysis across brain states, genotypes and sex.*** *Left most column contains description of test statistic and measurements analysed. Columns 2–4 contain relevant F, DF and P values for the effects of genotype and sex, and interaction effect between them. Values in rows 3–11 correspond to results from Two-way ANOVA test statistics and values in rows 14–16 correspond to results from Linear mixed models used in hour-by-hour time course analysis of sleep-wake distribution. Significant P values are highlighted in bold text and grey background. Statistical results relate to Supplementary Fig. 7.*


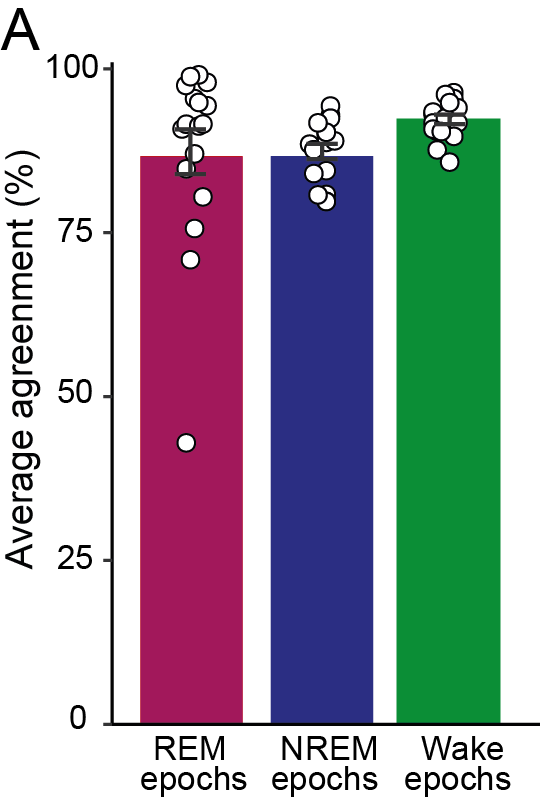


***Supplementary Figure 1 Percentage Agreement Between Sleep-Wake Automated Scoring Algorithm and Visual Scoring.***  *Agreement between visually and automatically scored epochs of REM, NREM and wake states is 88.1%, 87.5% and 95% respectively. Overall agreement between the scoring methods is 90.8%. The kappa coefficient was 0.83 (± 0.002 SE). See also Supplementary Table 1 for further details. Bars indicate mean values (mean ± SEM). Points correspond to values from individual rats.*


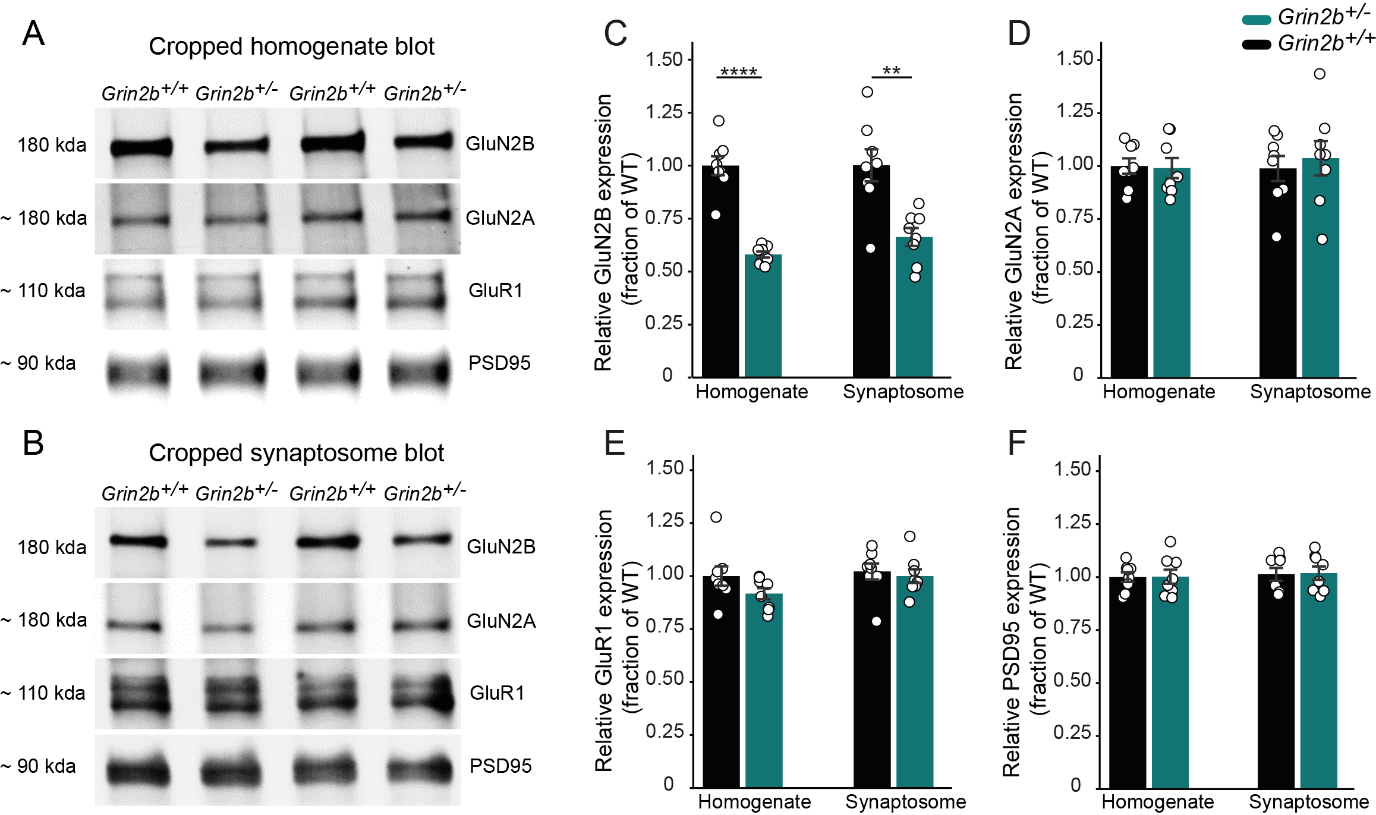


***Supplementary Figure 2 Grin2b deletion in rats results in reduction of endogenous GluN2B expression in hippocampus.*** *Representative Western blots of extracts from rat hippocampal brain (****A****) homogenates and (****B****) synaptosomes, full length blots in Supplementary Fig 3. Bands in the molecular weight range expected for full length GluN2B, GluN2A, GluR1 and PSD95 were detected in homogenates and synaptosomes from wild-type and Grin2b^+/-^ animals. (****C****) Quantification of GluN2B protein from homogenates and synaptosomes reveals a significant decrease in Grin2b^+/-^ rats (homogenate, Two-sample unpaired t-test, DF = 14, T = -897, P < 0.00001; synaptosome, Two-sample unpaired t-test, DF = 14, T = -3.89, P = 0.0016). There was no change in Grin2b^+/-^ rats in expression levels of (****D****) GluN2A (homogenate, Two-sample unpaired t-test, DF = 14, T = -0.16, P = 0.88; synaptosome, Two-sample unpaired t-test, DF = 14, T = 0.48, P = 0.64), (****E****) GluR1 (homogenate, Two-sample unpaired t-test, DF = 14, T = -1.57, P = 0.14; synaptosome, Two-sample unpaired t-test, DF = 14, T = -0.44, P = 0.67), and (****F****) PSD95 (homogenate, Two-sample unpaired t-test, DF = 14, T = 0.049, P = 0.96; synaptosome, Wilcoxon rank sum test, W = 28, P = 0.71). Bars indicate mean values (mean ± SEM). Points correspond to values from individual rats (n_+/+_ = 8, n_+/-_ = 8).*


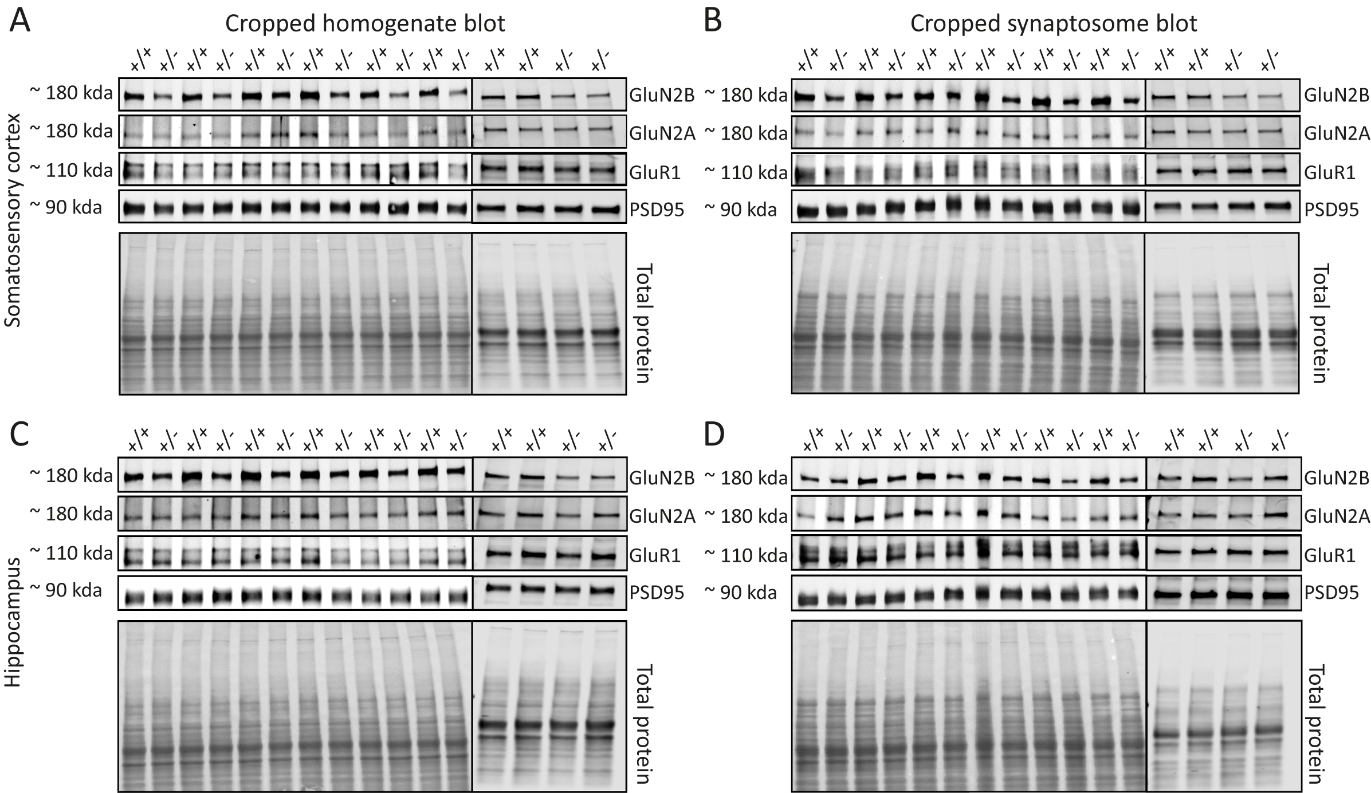


***Supplementary Figure 3 Grin2b deletion in rats results in reduction of endogenous GluN2B expression in somatosensory cortex and hippocampus.*** *Entire set of Western blots of extracts from rat somatosensory brain (****A****) homogenates and (****B****) synaptosomes, and extracts from rat hippocampal brain (****C****) homogenates and (****D****) synaptosomes. Bands in the molecular weight range expected for full length GluN2B, GluN2A, GluR1 and PSD95 were detected in homogenates and synaptosomes from wild-type and Grin2b^+/-^ animals.*


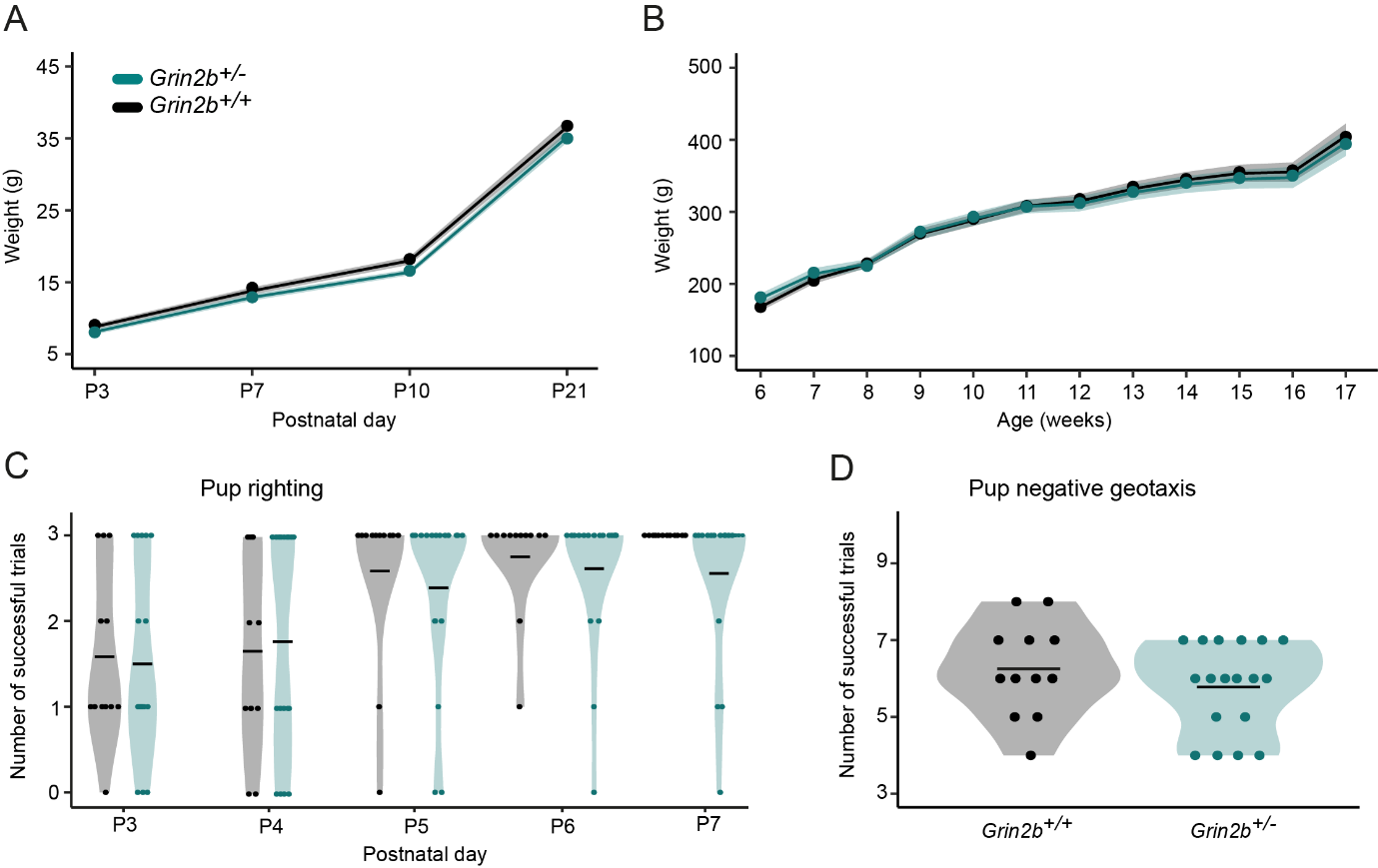


***Supplementary Figure 4 No differences in weight and motor development of pup and adult Grin2b^+/-^ and wild-type rats.*** *(****A****) Body weight of Grin2b^+/-^ pups from P3 to P21 did not differ from that of Grin2b^+/+^* *pup littermates (Linear Mixed Model, effect of genotype F = 2.78, DF = 1, P = 0.12; effect of age F = 1769.28, DF = 3, P < 0.00001; genotype x age F = 0.54, DF = 3, P = 0.66) (n_+/+_ = 5, n_+/-_ = 8). (****B****) Similarly, no genotype related differences were observed in adult body weight between 6 and 17 weeks of age (Linear Mixed Model, effect of genotype F = 0.0056, DF = 1, P = 0.94; effect of age F = 715.58, DF = 11, P < 0.00001; genotype x age F = 0.86, DF = 11, P = 0.58) (n_+/+_ = 47, n_+/-_ = 49). Points indicate mean values for all animals (mean ± SEM). (****C****) Grin2b⁺^/^⁻ and Grin2b⁺^/^⁺ pups exhibited comparable performance in the righting reflex task across developmental days, with righting reflex performance improving significantly over the measured developmental period (Linear Mixed Model, effect of genotype F = 0.29, DF = 1, P = 0.6; effect of age F = 18.36, DF = 4, P < 0.00001; genotype x age F = 0.57, DF = 4, P = 0.68) (n_+/+_ = 12, n_+/-_ = 18). (****D****) No differences were detected between genotypes on negative geotaxis performance in pups* between P7 and P10 *(Wilcoxon rank sum test, W = 87, P = 0.37) (n_+/+_ = 12, n_+/-_ = 18). Points indicate individual animals. Shaded areas represent the distribution of the data with mean values for each group.*


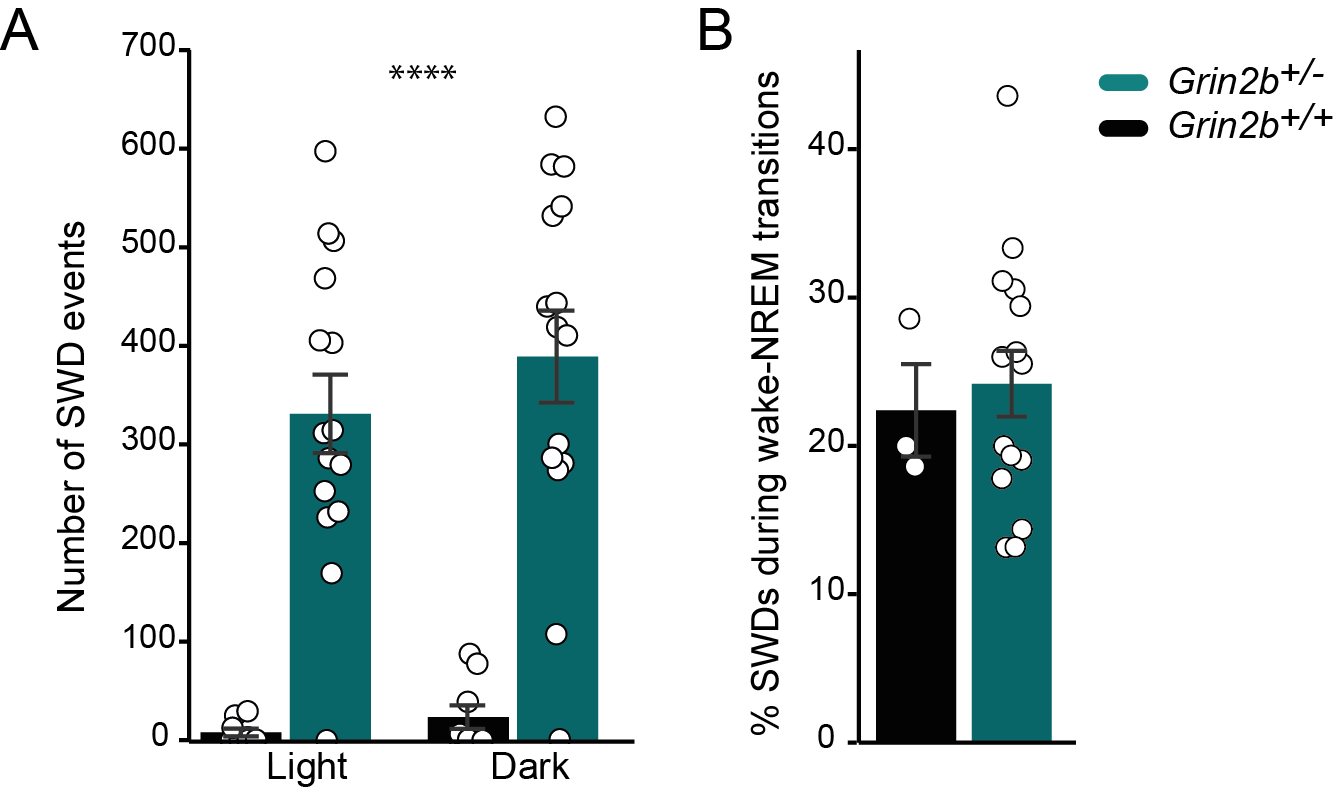


***Supplementary Figure 5Light phase did not influence the prevalence of SWDs and the percentage of wake-NREM transitions did not differ between genotypes.*** *(****A****) Number of SWD events during light and dark phases did not significantly differ between light and dark phases but were significantly increased in Grin2b^+/-^ rats (Linear Mixed Model, effect of genotype F = 55.36, DF = 1, P < 0.00001; effect of phase F = 1.23, DF = 1, P = 0.28; phase x genotype F = 0.41, DF = 1, P = 0.53), (*** = effect of genotype). Grin2b^+/-^ rats showed similar SWD event numbers in both light and dark periods, which significantly exceeded SWD amounts in wild-type littermates during both phases (effect of genotype P < 0.00001; effect of phase P = 0.28; phase x genotype P = 0.53, Linear Mixed Model) (n_+/+_ = 9, n_+/-_ = 15). Bars indicate mean values (mean ± SEM). (****B****) A higher proportion of SWDs in NREM initiate during wake-NREM transitions in Grin2b^+/-^ animals when compared to wild-type animals (Two-sample unpaired t-test, DF = 16, T = -0.34, P = 0.74). Amount of SWDs initiating at wake-NREM transitions were not different between Grin2b^+/-^ rats and Grin2b^+/+^ wild-types that did have SWDs at transitional periods (3/9 animals) (P = 0.74, Two-sample unpaired t-test) (n_+/+_ = 3, n_+/-_ = 15). Bars indicate mean values (mean ± SEM) and points correspond to values from individual rats.*


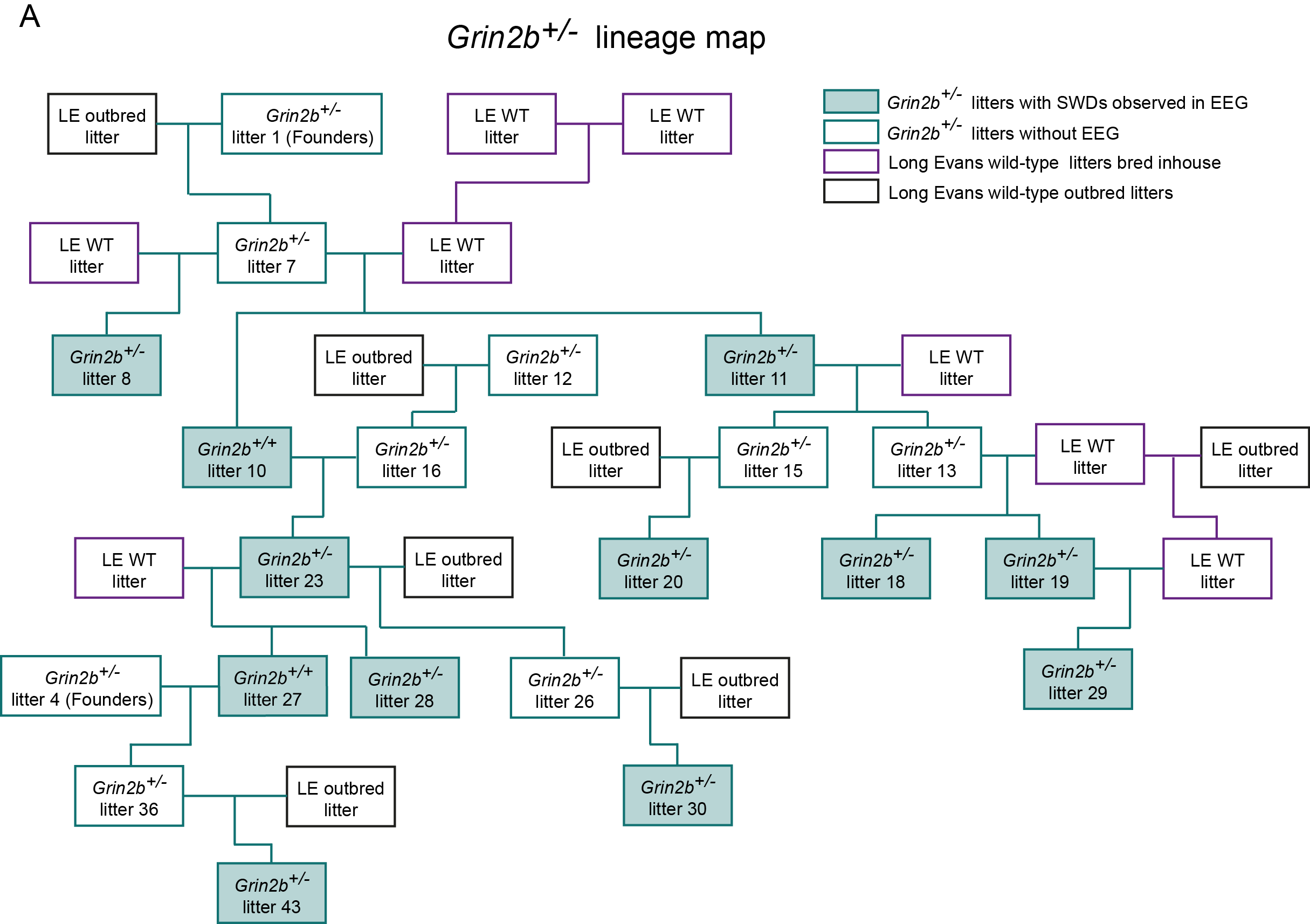


***Supplementary Figure 6 Lineage diagram of Grin2b⁺^/^⁻ rats.*** *(****A****) Diagram showing the breeding lineage of Grin2b⁺^/^⁻ rats across multiple generations. Teal boxes represent Grin2b colony litters, with Grin2b⁺^/^⁻ and Grin2b⁺^/^⁺ animals used for breeding or experimental purposes. Teal filled boxes indicate litters in which spontaneous SWDs were detected through EEG recording. Teal outlined boxes represent Grin2b litters that were not assessed with EEG but were used for breeding. Black outlined boxes denote Long Evans wild-type outbred litters, while purple outlined boxes indicate wild-type litters bred in-house. This schematic illustrates the persistence of the SWD phenotype across multiple generations.*


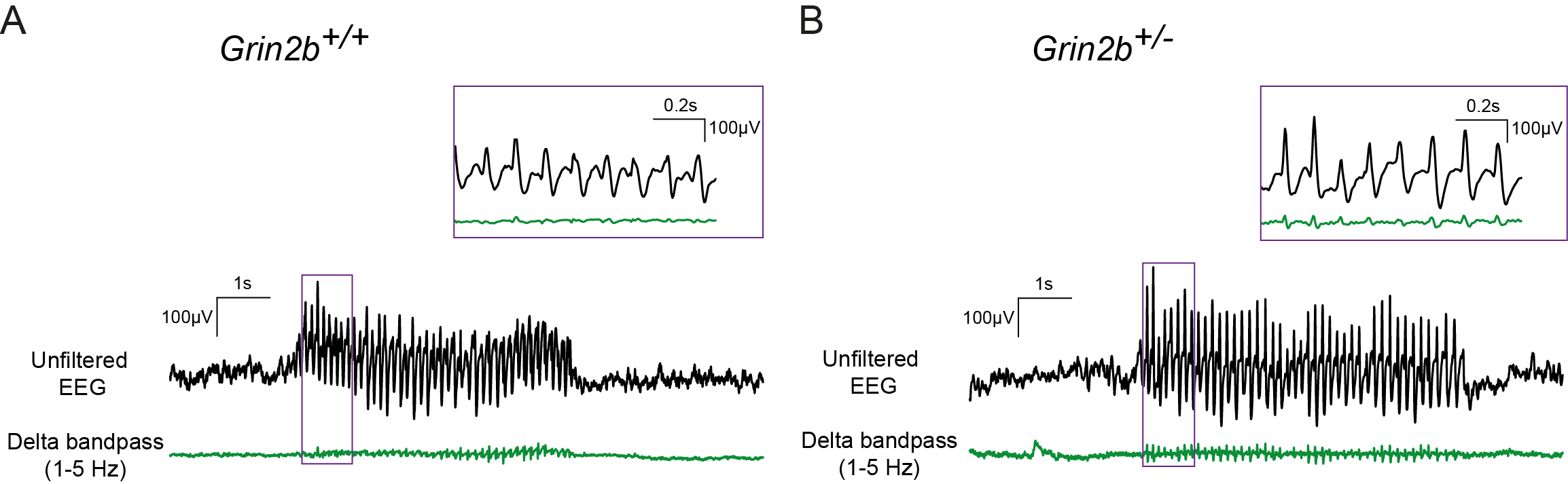


***Supplementary Figure 7 Increased SWD delta power amplitude in Grin2b^+/-^ rats.*** *(****A****) Representative EEG recording from a Grin2b^+/+^* during *a SWD event, showing the raw unfiltered voltage trace (black) alongside the delta bandpass-filtered trace (green).* (***B****) Corresponding EEG recording from a Grin2b^+/−^ rat during an SWD, with raw unfiltered (black) and delta-filtered (green) signals.* *Insets show expanded views of the boxed regions, highlighting SWD morphology* and associated delta oscillations, which are of larger amplitude in *Grin2b^+/−^ rats (see main Fig. 2 and Results section).*


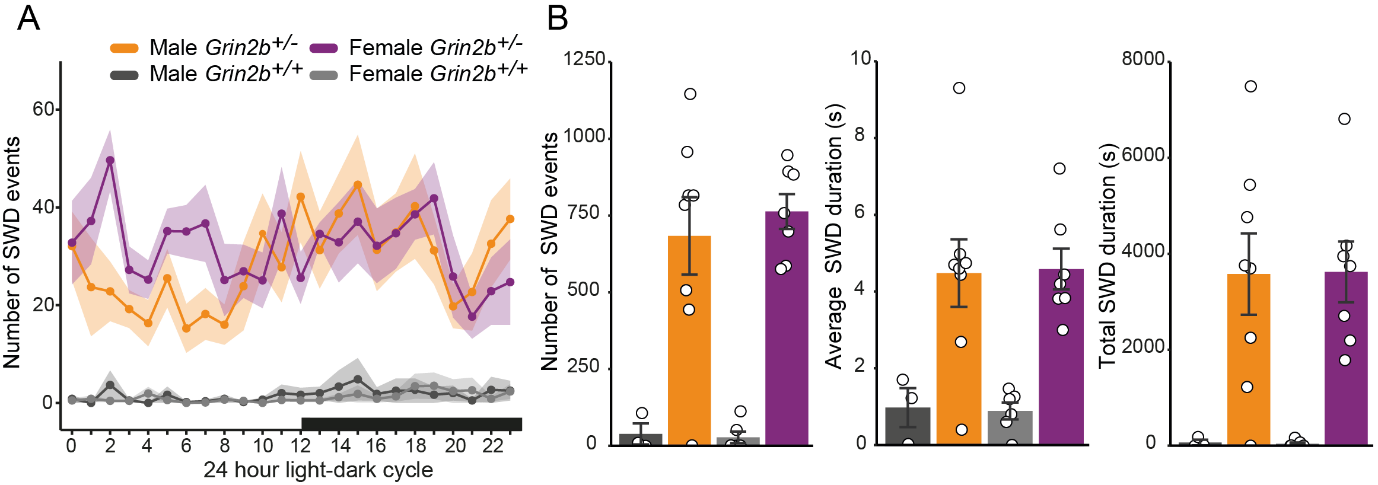


***Supplementary Figure 8 differences in*** ***SWD properties between male and female Grin2b^+/-^ rats.*** *(****A****) Number of SWD events plotted by hour over the 24-hour light-dark cycle with black bar on x-axis indicating lights off in the animal facility.* *Hour of the day and sex do not impact on the amount of SWDs in Grin2b^+/-^ male and female rats, which consistently had significantly more seizures than wild-type sex-matched littermates (Linear Mixed Model, effect of hour F = 1.24, DF = 23, P = 0.21; genotype F = 47.83, DF = 1, P < 0.00001; sex F = 0.12, DF = 1, P = 0.73, hour x genotype x sex F = 0.85, DF = 23, P = 0.66) ) (male n_+/+_ = 3, female n_+/+_ = 6, male n_+/-_ = 8, female n_+/-_ = 7). Points indicate mean values of all animals (mean ± SEM). (****B****) Bar plots of total number of SWDs events (left), SWD average duration (middle) and total SWD duration (right). Grin2b^+/-^ rats had a statistically greater number of SWDs, with longer average and total durations than Grin2b^+/+^ rats, and these differences were not influenced by the animals’ sex (SWD number (Two-way ANOVA, effect of sex F = 0.12, DF = 1, P = 0.74; genotype F = 47.663 DF = 1, P < 0.0001; sex x genotype F = 0.2, DF = 1, P = 0.66), SWD average duration (Two-way ANOVA, effect of sex F = 0.0002, DF = 1, P = 0.99; genotype F = 23.17, DF = 1, P = 0.0001; sex x genotype F = 0.017, DF = 1, P = 0.89), total SWD duration (Two-way ANOVA, effect of sex F = 0.0001, DF = 1, P = 0.99; genotype F = 22.9, DF = 1, P = 0.0001; sex x genotype F = 0.002, DF = 1, P = 0.96). Bars indicate mean values (mean ± SEM). Points correspond to values from individual rats (male n_+/+_ = 3, female n_+/+_ = 6, male n_+/-_ = 8, female n_+/-_ = 7). Note: we do not indicate significance (*) where effect from genotype is found.*


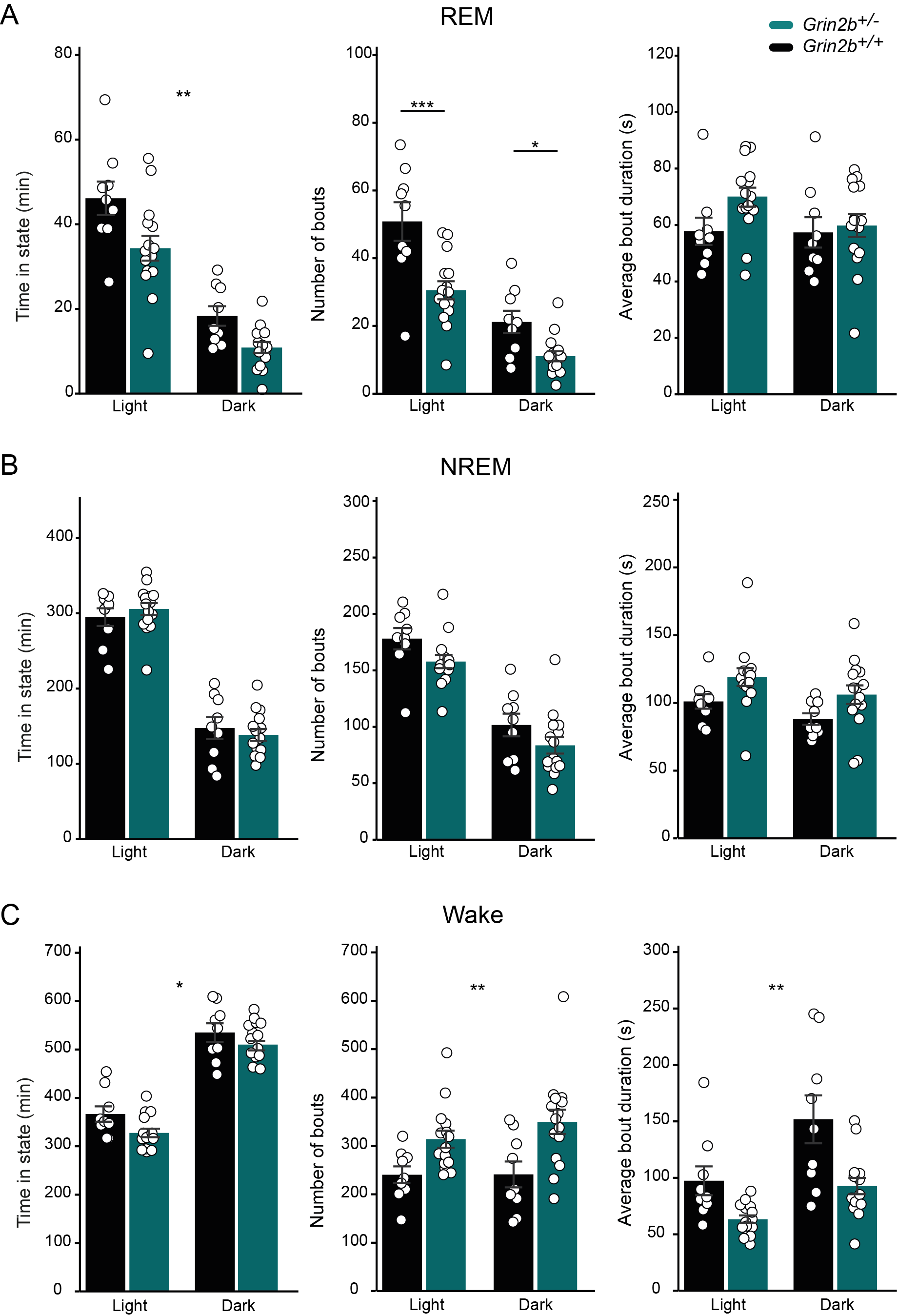


***Supplementary Figure 9 Reduced REM sleep in Grin2b^+/-^ rats during the light and dark periods.*** *Quantification showing total time (left), number of bouts (middle) and average bout duration (right) for (****A****) REM, (****B****) NREM and (****C****) wake during the 12-hour light and dark periods. Total time in REM sleep was overall reduced in Grin2b^+/-^ rats compared to wild-types, and reduced REM sleep was not specific to either light or dark phases (Linear Mixed Model, effect of phase F = 119.42, DF = 1, P < 0.00001; genotype F = 9.99, DF = 1, P = 0.0046; phase x genotype F = 0.85, DF = 1, P = 0.37). (** = effect of genotype). In comparison to Grin2b^+/+^ animals, the number of REM bouts in Grin2b^+/-^ rats was reduced, both during the light and the dark periods (Linear Mixed Model, effect of phase F = 105.93, DF = 1, P < 0.00001; genotype F = 15.52, DF = 1, P = 0.00067; phase x genotype F = 4.53, DF = 1, P = 0.045; Tukey post hoc test, light P = 0.0001, dark P = 0.032), while no differences were found between the two groups in the average length of REM bouts (Linear Mixed Model, effect of phase F = 2.26, DF = 1, P = 0.13; genotype F = 1.88, DF = 1, P = 0.18; phase x genotype F = 2.15, DF = 1, P = 0.16). Analysis by light-dark phase revealed no difference between the two genotypes for total time spent in NREM sleep (Linear Mixed Model, effect of phase F = 237.34, DF = 1, P < 0.00001; genotype F = 0.0065, DF = 1, P = 0.94; phase x genotype F = 0.94, DF = 1, P = 0.34), the number of NREM bouts (Linear Mixed Model, effect of phase F = 198.28, DF = 1, P < 0.00001; genotype F = 3.62, DF = 1, P = 0.07; phase x genotype F = 0.041, DF = 1, P = 0.84) and the average NREM bout durations (Linear Mixed Model, effect of phase F = 16.34, DF = 1, P = 0.00055; genotype F = 4.091, DF = 1, P = 0.055; phase x genotype F = 0.0023, DF = 1, P = 0.99). Differences between Grin2b^+/-^ rats and littermate controls in total wake minutes were observed and these differences were not influenced by light or dark phases (Linear Mixed Model, effect of phase F = 185.12, DF = 1, P < 0.00001; genotype F = 6.64, DF = 1, P = 0.013; phase x genotype F = 0.23, DF = 1, P = 0.64), wake bouts (Linear Mixed Model, effect of phase F = 1.39, DF = 1, P = 0.25; genotype F = 9.76, DF = 1, P = 0.0049; phase x genotype F = 1.27, DF = 1, P = 0.27) or average wake bout duration (Linear Mixed Model, effect of phase F = 26.25, DF = 1, P < 0.00001; genotype F = 13.14, DF = 1, P = 0.0015; phase x genotype F = 2.34, DF = 1, P = 0.14). (* and ** = effect of genotype). Bars indicate mean values (mean ± SEM) and points correspond to values from individual rats (n_+/+_ = 9, n_+/-_ = 15).*


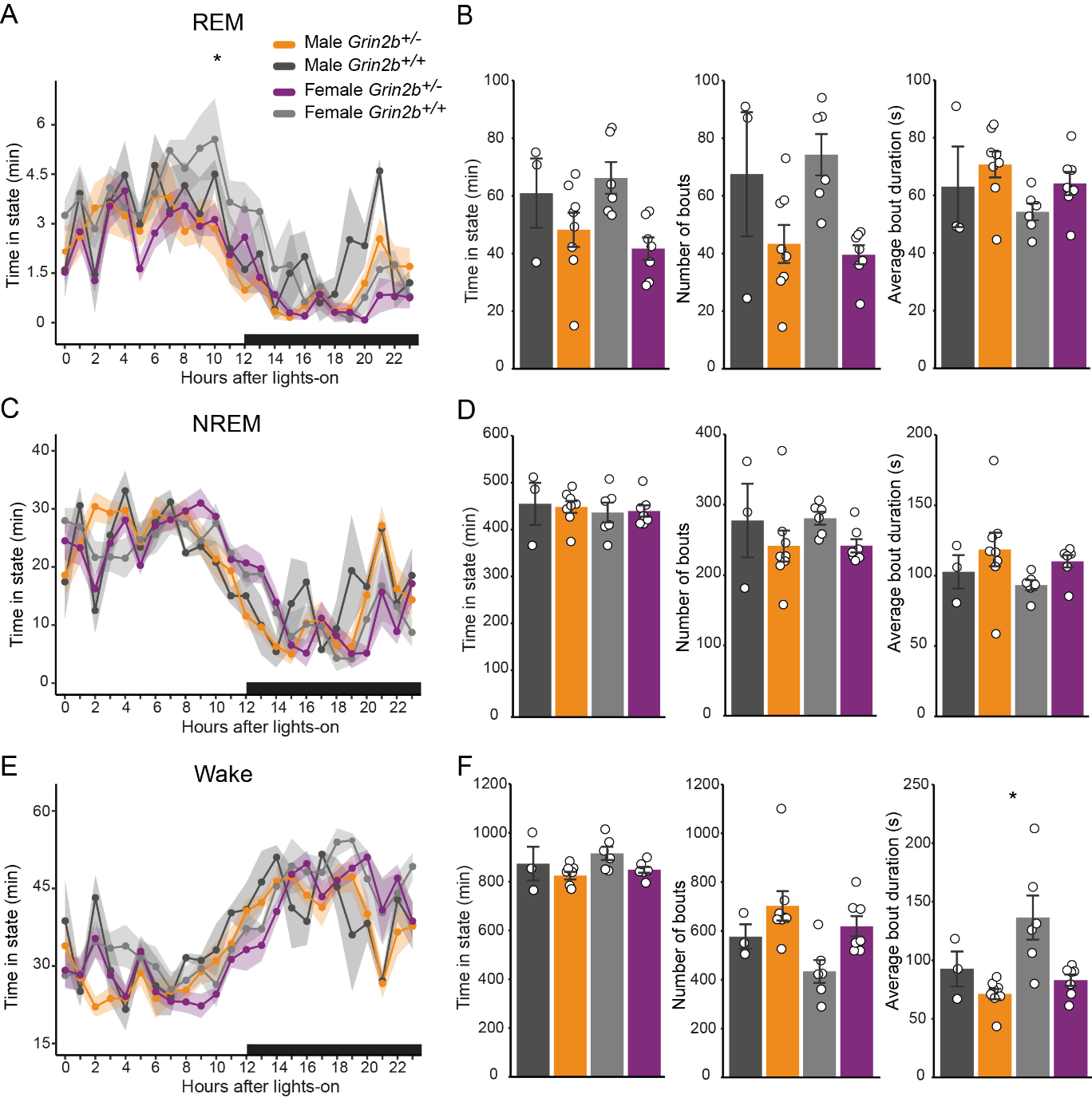


***Supplementary Figure 10 Sleep-wake physiology in Grin2b^+/-^ animals does not differ between male and female animals.*** *Quantification showing the time spent in (****A****) REM, (****C****) NREM and (****E****) wake by hour across the 24-hour light-dark cycle; points indicate mean values of all animals (mean ± SEM). Bar plots showing total time (left), number of bouts (middle) and average bout duration (right) for (****B****) REM, (****D****) NREM and (****F****) wake during the full 24 hours; bars indicate mean values (mean ± SEM) and points correspond to values from individual rats. (****A****) Time in REM is reduced in both female and male Grin2b^+/-^ rats relative to female and male wild-type littermates, and overall, female rats spent slightly longer in REM sleep than male animals. These differences were not influenced by specific hours of the day (* = effect of sex). (****B****) For the full 24 hours, however, sex did not impact on total REM sleep or REM bouts, as each was reduced in both male and female Grin2b^+/-^ mutants relative to their wild-type sex-matched littermates. Average REM bout duration was not different between both sexes and genotypes. (****C****) Time in NREM is similar between Grin2b^+/-^ and wild-type animals of both sexes throughout each hour of the day. (****D****) For the 24-hour day, sex did not impact on total NREM sleep, NREM bouts and average NREM bout duration, and these metrics did not differ between male and female Grin2b^+/-^ and Grin2b^+/+^ animals. (****E****) Time in wake is reduced in both female and male Grin2b^+/-^ rats relative to female and male Grin2b^+/+^ littermates, and this difference is not due to animals’ sex or to specific hours of the day. (****F****) There we no sex dependent differences for the total wake time and number of wake bouts when quantified for the full 24 hours, while average wake bout duration was longer in female rats than in males (* = effect of sex). For detailed statistics see Supplementary Table 2. Note: we do not indicate significance (*) where effect from genotype is found. Bars indicate mean values (mean ± SEM). Points correspond to values from individual rats (male n_+/+_ = 3, female n_+/+_ = 6, male n_+/-_ = 8, female n_+/-_ = 7).*


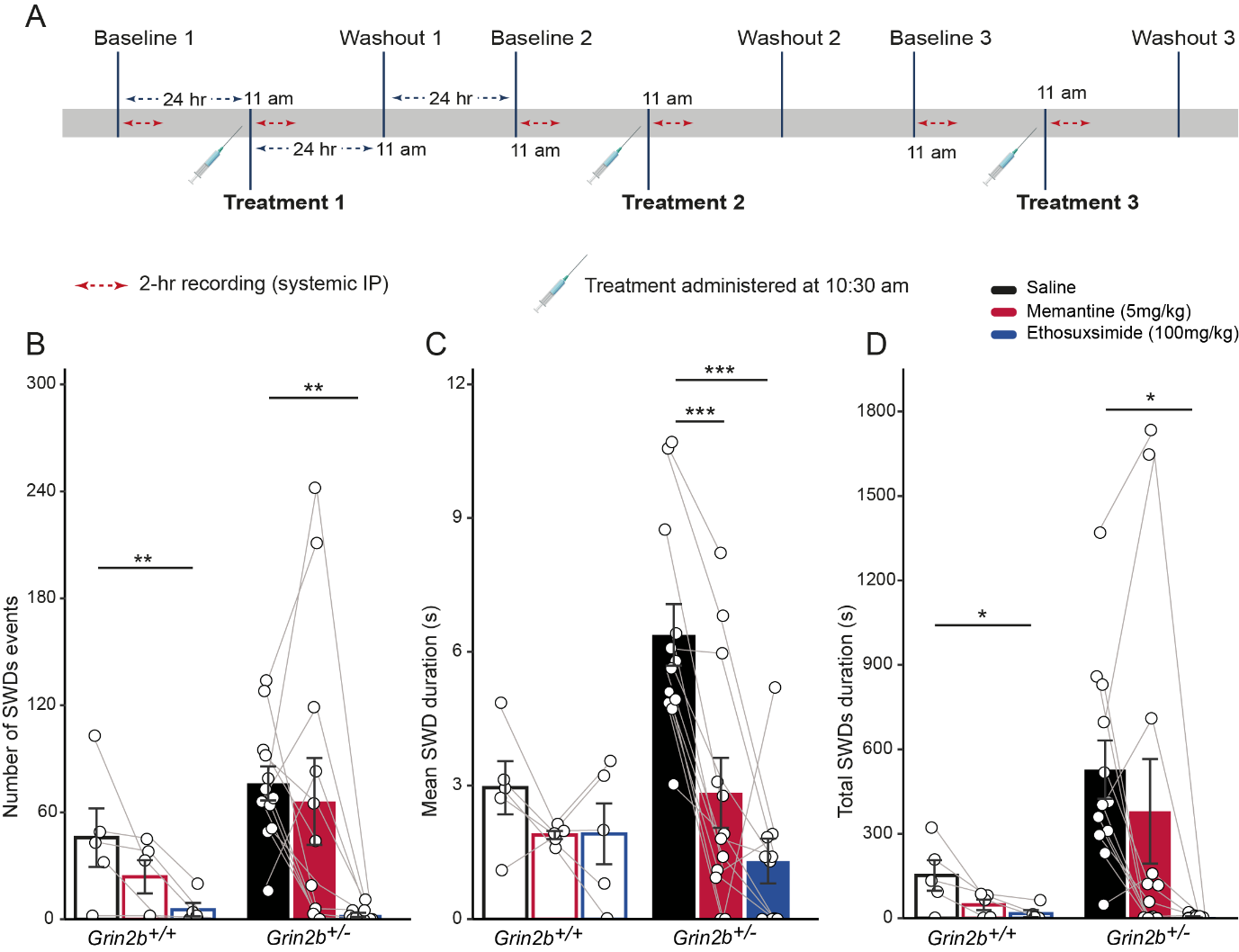


***Supplementary Figure 11 Acute administration of anti-seizure drugs attenuates SWDs in Grin2b^+/-^ and wild-type animals - absolute value analysis.*** *(****A****) Diagram of pharmacology experiment timeline. Note: Treatment order is randomized and weighted across animals to minimize the risk of treatment carry-over effects. Plots showing effects of acute drug treatment on SWD number (****B****), average duration (****C****) and total duration (****D****) of SWDs in Grin2b^+/-^ and wild-type animals. Data are presented as absolute values (i.e., not normalized to baseline). Treatment with ethosuximide blocked SWD events in both Grin2b^+/-^ and Grin2b^+/+^ rats (Linear Mixed Model, effect of treatment F = 5.69, DF = 2, P = 0.0091; genotype F = 2.08, DF = 1, P = 0.18; treatment x genotype F = 0.84, DF = 2, P = 0.44; Tukey post hoc test, ethosuximide-saline P = 0.0074, ethosuximide-memantine P = 0.066, memantine saline P = 0.61). Memantine and ethosuximide decreased the average duration of SWDs in Grin2b^+/-^ rats, while no significant effect observed in wild-type controls (Linear Mixed Model, effect of treatment F = 15.16, DF = 2, P < 0.0001; genotype F = 2.19, DF = 1, P = 0.16; treatment x genotype F = 5.65, DF = 2, P = 0.0089; Tukey post hoc test, Grin2b^+/-^ ethosuximide-saline P < 0.0001, Grin2b^+/-^ ethosuximide-memantine P = 0.2, Grin2b^+/-^ memantine saline P < 0.0001, Grin2b^+/+^ ethosuximide-saline P = 0.5, Grin2b^+/+^ ethosuximide-memantine P = 0.99, Grin2b^+/+^ memantine-saline P = 0.48). Ultimately, ethosuximide led to a significant reduction in total SWD duration in both Grin2b^+/-^ and wild-type animals (Linear Mixed Model, effect of treatment F = 3.85, DF = 2, P = 0.034; genotype F = 2.28, DF = 1, P = 0.16; treatment x genotype F = 1.28, DF = 2, P = 0.29; Tukey post hoc test, ethosuximide-saline P = 0.025, ethosuximide-memantine P = 0.28, memantine-saline P = 0.45). Bars indicate mean values (mean ± SEM). Points correspond to values from individual rats and grey lines follow treatment response of each individual rat (n_+/+_ = 5, n_+/-_ = 12).*


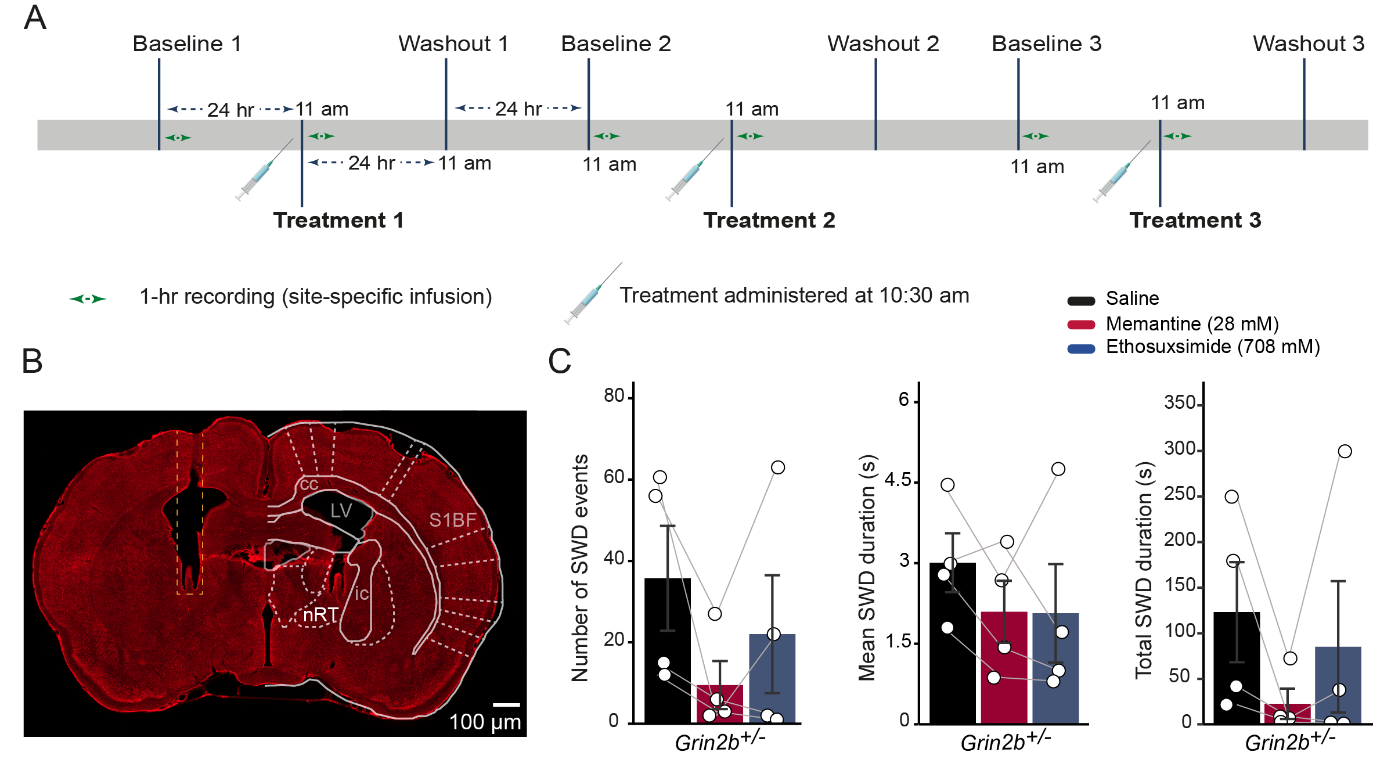


***Supplementary Figure 12 Effects of site-specific infusion of anti-seizure drugs in nRT on SWDs in Grin2b^+/-^ rats - absolute value analysis.*** *(****A****) Diagram of pharmacology experiment timeline. Note: Treatments order is randomized and weighted across animals to minimize the risk of treatment carry-over effects (****B****) Representative histology image showing bilaterally implanted cannulas in the rostral nRT (yellow dashed lines), overlaid with approximate coordinates from Paxinos and Watson’s Rat Brain Atlas* (Paxinos & Watson, 2018)*. (****C****) Panels showing effects of acute nRT drug infusion on SWD average number (left), average duration (middle) and total duration (right) of SWDs in Grin2b^+/-^ rats. Treatment with ethosuximide and memantine does not block SWD events in Grin2b^+/-^ animals relative to saline control treatment (Linear Mixed Model, effect of treatment F = 3.33, DF = 2, P = 0.11). Neither drug reduces average SWD durations (Linear Mixed Model, effect of treatment F = 1.67, DF = 2, P = 0.27) or total seizure durations (Linear Mixed Model, effect of treatment F = 2.38, DF = 2, P = 0.17) compared to saline in Grin2b^+/-^ rats. S1BF = primary somatosensory cortex - barrel field, nRT = reticular thalamic nucleus, LV = lateral ventricle, cc = corpus callosum, ic = internal capsule. Bars indicate mean values (mean ± SEM). Points correspond to values from individual rats and grey lines follow treatment response of each individual rat (n_+/-_ = 4).*

**Supplementary Methods**

**Animals**

Pups were bred in-house and weaned from their dams at postnatal day 22. They were subsequently housed in mixed genotype cages with wild-type littermates (two to six rats per cage) with a 12-hour/12-hour light-dark cycle with ad libitum access to water and food. Following surgery, animals were single housed. Rats were genotyped by polymerase chain reaction.

**Synaptosome preparation**

Adult 19-week-old *Grin2b^+/-^* (six male, two female) and *Grin2b^+/+^* (three male, five female) rats were anesthetised with isoflurane and decapitated. Homogenate and synaptosome fractions from hippocampus and somatosensory cortex tissue were prepared.

The hippocampus and somatosensory cortex from each hemisphere were quickly dissected in slushy PBS 1X, snap frozen and stored at -80 °C. The discontinuous Percoll-density gradient (23% bottom, 10% middle, 3% uppermost) was made prior to homogenisation on the preparation day. The tissue was quickly thawed at 37 °C and homogenized in ice-cold homogenisation buffer (1x sucrose/EDTA, pH7.4) using 5-6 up-and-down strokes of a pre-chilled Teflon glass with a motorized homogenizer^1^ followed by centrifugation at 2800 rpm for 10 min at 4°C. The supernatant was added gently on 3% Percoll-sucrose (Percoll, P1644, Sigma Aldrich, UK) and centrifuged at 20,000 rpm for 8 min at 4°C. The fraction between 23% and 10% was collected and re-suspended in HEPES-Buffered-Krebs (HBK; in mM: 118.5 NaCl, 4.7 KCl, 1.18 MgSO_4_, 10 Glucose, 1 Na2HPO_4_, 20 HEPES, pH 7.4 balanced with Trizma) followed by centrifugation at 13,000 rpm for 15 min at 4°C. The pellet (pure synaptosomes) was dissolved in RIPA buffer with protease inhibitor cocktail (5056489001, Roche, Switzerland) and phosphatase inhibitor cocktails II and III, (P5726 and P0044 respectively, Sigma Aldrich, United Kingdom). Protein concentration was determined with the MicroBCA Assay kit (Pierce BCA protein estimation kit, 23225, ThermoFisher Scientific, United Kingdom).

**Growth and motor function assessment**

Eight *Grin2b^+/-^* and five *Grin2b^+/+^* pups were weighed once per week at postnatal day three (P3), P7, P10 and P21. Natural coat marking patterns were used to identify pups before ear notching at weaning. Rats were weighed weekly, from six to seventeen weeks of age.

Pup righting reflex was tested daily from P3 to P7. Individual pups were identified across days using natural coat coloration patterns documented in photographs prior to testing. Eighteen *Grin2b^+/-^* and twelve *Grin2b^+/+^* pups were gently removed from their home cage and placed in a clean holding cage on a heat pad for the duration of the test. Each pup underwent three 15 second trials per day, with 20 second intervals between trials, during which the pup was held in a nitrile-gloved hand to maintain body temperature. For each trial, pups were placed in the supine position and held gently for 2–3 seconds before release. The latency to return to a prone position (i.e. all four paws on the ground) was recorded as the righting latency, with a maximum trial duration of 15 seconds. 'Righting ability' was defined as successful completion of this posture within the trial window. 'Righting success' was defined as achieving righting ability in at least one of three trials per day on two consecutive days. The second consecutive day of successful righting was recorded as the day the pup reached criterion.

Negative geotaxis was assessed daily from P3 to P10 in the same cohort of eighteen *Grin2b^+/-^* and twelve *Grin2b^+/+^* pups, approximately 30 minutes following completion of the righting reflex task. Between tasks, pups were kept in a holding cage on a heat pad. A wooden plank measuring 30 × 70 cm was positioned at a 30° incline. The plank was covered with an aluminum perforated metal sheet (RS 202-7906, 2 mm holes; RS PRO, UK), marked with pen lines indicating the target angle pups were required to reach to successfully complete the trial. Pups were individually placed on the inclined plank with their heads oriented downward (180°) and all four paws fully in contact with the surface. Upon release, pups were allowed to attempt to turn and orient themselves facing upward along the slope. If a pup fell during the trial, it was immediately caught and gently returned to the starting position (180°). This procedure was repeated until pups either achieved a successful turn, accumulated nine falls, or 45 seconds elapsed, whichever occurred first. Successful turning was defined as the pup’s head orienting within a 60° window centered on the upward direction (0°), encompassing 30° to either side. To reach criterion, pups were required to successfully turn in at least one attempt out of ten trials on two consecutive days. Trials in which pups failed to achieve this orientation within the allotted attempts/ time were recorded as unsuccessful. The number of successful attempts was recorded for analysis.

**Western Blot**

Approximately 15 μg of synaptosome protein was separated on a precast gradient gel (4-15% Mini-PROTEAN® TGX™ Precast Protein Gels, 4561086, BioRad, United Kingdom) and transferred to nitrocellulose membrane (Amersham™ Protran® Western Blotting Membrane, Nitrocellulose, GE10600002, Sigma Aldrich, United Kingdom) using a Bio-Rad transfer apparatus. Total proteins were stained with a reversible protein stain kit (Memcode 24580, Thermo Fisher Scientific, United Kingdom) according to the manufacturer’s instructions. After removing the stain, membranes were blocked with 1:1 TBS1X: Odyssey Blocking Buffer (P/N-927-50003, LI-COR Biotech.) for an hour at room temperature, followed by overnight incubation with primary antibodies (NMDAR2A-1: 1000, #ab169873, Abcam; NMDAR2B- 1:1000, #610417, BD Biosciences; PSD95- 1:2000, #76115, Abcam; GluR1- 1:1000, #MAB2263, Millipore) at 4°C. Membranes were washed with TBST1X (0.1% Tween 20), and incubated for an hour at room temperature with secondary antibodies (IRDye 680RD Donkey anti-rabbit IgG- 1:5,000, #P/N 926-68073; IRDye 800CW Donkey anti-mouse IgG - 1:5,000, #P/N 926-32212, LI-COR Biotechnology, United Kingdom). Membranes were washed with TBST1X, dried and digitally scanned using an Odyssey M Imager, LI-COR, UK Ltd. Odyssey software, Licor Image Studio Lite (LICOR Biosciences, United Kingdom) was used to quantify individual bands. Data was normalised to respective total protein and normalised to wild-type levels.

**Surgery**

Adult rats were anaesthetised with isoflurane and mounted on a stereotaxic frame (David Kopf Instruments, United States). Animals received subcutaneous Rimadyl Small Animal Solution (5 mg/kg, Zoetis, United states) for analgesia and 2.5 ml of sterile 0.9% saline for rehydration.

For surface EEG grid implantation, two holding screws for structural support (4 mm rostral, ± 0.5 mm lateral relative to bregma) and one ground screw over the cerebellum (-11.5 mm rostral, 0.5 mm lateral relative to bregma) (Yahata Neji, M1 Pan Head Stainless Steel Cross, RS Components, United Kingdom) were attached. 16-channel EEG surface grids with two integrated electromyogram (EMG) leads (Custom H16-Rat EEG16_Functional-NeuroNexus, United States) were placed over the skull with the EEG surface grid plus-symbol reference point aligned over bregma. Silver paint was used for connecting the ground screw with the grid. The implant was fixed to the skull using dental cement (Simplex Rapid, Kemdent, United Kingdom). A 21 G sterile needle tip was used to guide EMG wires (2.5 cm in length with 0.5 cm striped of insulation) into the neck muscle, after which the incision was sutured. Fifteen 16–9 week-old *Grin2b^+/-^* (eight male, seven female) and nine *Grin2b^+/+^* (three male, six female) animals were implanted, recorded and used for analyses. Four *Grin2b^+/-^* and nine *Grin2b^+/+^* rats that were descendants of one specific wild-type founder rat were excluded as they exhibited higher rates of absence seizures than descendants from other founders, which obfuscated genotype differences. Wild-type animals descended from the specific founder displayed between 1 and 1117 SWDs in 24 hours, on average 481 seizures over the rest of the population. Our current *Grin2b^+/+^* rats (decedents from multiple founders) do not display such a high prevalence of SWDs. Sample size estimates were not performed as these were the first recordings from this rat line.

For 2-channel EEG recordings for pharmacology experiments, bilateral screws were implanted to provide structural support (4 mm rostral, ± 0.5 mm lateral relative to bregma). Two ground screws were attached over the cerebellum (-11.5 mm rostral, ± 0.5 mm lateral relative to bregma), and two bilateral electrode screws were fixed over the primary somatosensory cortex (- 2.5 mm rostral, ± 4.5 lateral relative to bregma) (Yahata Neji, M1 Pan Head Stainless Steel Cross, RS Components). Electrode and ground screws were connected to an electronic interface board (EIB-16, Neuralynx) via silver wire soldered to each component (Teflon Coated Silver Wire, World Precision Instruments). The implant was fixed to the skull using dental cement (Simplex Rapid, Kemdent, United Kingdom). Twelve 21–26 week-old *Grin2b^+/-^* (ten male, two female) and five *Grin2b^+/+^* (one male, four female) animals were recorded and used in pharmacology experiments. A further four *Grin2b^+/+^* rats were implanted and recorded, but were excluded from pharmacological experiments as they did not display seizures during baseline recording days.

Site specific drug infusions were conducted specifically on *Grin2b^+/-^* rats. For drug infusion cannula implantation, two craniotomies were drilled bilaterally over the rostral reticular thalamic nucleus (nRT) (- 1.45 mm rostral, ± 2.2 lateral relative to bregma). The dura was removed from each craniotomy and a single 26 G sterile guide cannula (diameter = 0.46 mm, P1 Technologies) was slowly inserted to target the nRT (-5 mm ventral from the brain surface). Guide cannulas were cemented in place using UV activated cement (3M Relyx Unicem 2 Automix, Henry Schein) and dummy cannulas were placed into guides to ensure guides remained contaminant and blockage free. EEG and EMG components were then implanted as described above (2-Channel EEG). Four 20 week-old *Grin2b^+/-^* animals were implanted and used in site specific drug infusion experiments. An additional *Grin2b^+/-^* rat was implanted with cannulas, but was excluded from the final drug infusion analysis as the animal did not display SWDs during recording days.

**Absence seizure detection and sleep-wake scoring**

As previously described, the SWD detection method consisted of the following: Spectral analysis revealed that visually scored SWDs behave as a high energy echo of a fundamental frequency (f0) located on the 5–10 Hz theta band, that resonates in several periodic harmonics across the frequency spectrum. This oscillating spectral structure of SWDs resembles a periodic waveform, allowing its automatic identification through a cepstral 1 analysis approach^2^ by searching for a high amplitude peak located on a frequency band of interest. We applied an automated SWD seizure detection algorithm to voltage traces from the EEG grid electrode lead overlaid approximately on primary somatosensory cortex (right hemisphere, AP -3.0 mm and ML 2.8 mm from bregma), as, by visual assessment, was the channel most frequently associated with high amplitude SWDs across animals. After deconvolving the raw signal using a Fast Fourier Transform (number of tapers = 5), a logarithm was applied to obtain the magnitude. The signal could then be treated as semi-periodic so that the inverse Fast Fourier Transform could be applied to obtain the cepstrum and reveal the period of the fundamental frequency (f0) as a spike in a pseudo-time domain frequency. After obtaining the cepstrum for the entire EEG recording (in sliding windows of 0.2 sec), peak power cepstrum values within the relevant frequency range (5–10 Hz) were identified and normalized by their absolute maximum. The resulting vector was transformed into z-scores to homogenise possible power differences between recordings that could distort seizure threshold identification. A threshold of ≥ 2.2 x 10^-5^ standard deviations was set by comparing the values of visually scored seizures against other high magnitude noise that resulted in false positives. 0.2 sec time windows were time-stamped as seizures when z-scored peak cepstral power in the theta band was greater than or equal to the established standard deviation threshold. The code used for analysis is available at Zenodo and Github: https://zenodo.org/records/12700972.

After SWD times were identified, a custom-made automated sleep scoring algorithm, based on a previously published method,^3^ which clusters EEG and EMG recording epochs into corresponding sleep-wake states based on the spectral power at specific frequency bands associated with those states, was used to assign epochs to one of three brain states: wake, rapid eye movement (REM) sleep and non-rapid eye movement (NREM) sleep. SWD times were excluded from this analysis. Traces from EEG channels overlying primary somatosensory cortex on either brain hemisphere and the two EMG channels were visually inspected for artefacts. The EEG and EMG channels with fewer artefacts were chosen for analysis and if both EEG or EMG traces had similar noise levels, one channel for each modality was randomly chosen for analysis. Only one animal was excluded due to complete signal loss due to poor grounding. Spectral logarithmic EEG and EMG power was calculated for non-overlapping 5 second epochs in the 0.2–125 Hz frequency range using the *multitaper* package^4^ for R^5^, using a non-overlapping window-size of 0.2 Hz half-time and bandwidth product of three. Each epoch was plotted in a three-dimensional metric space with values representing theta EEG power (maximum of the power in the 6.0 – 8.2 Hz range), delta and sigma power (1–20 Hz mean power, excluding the theta range), and neck muscle activity power (EMG power in the 60–90 Hz range). For clustering into brain states, centroids were estimated using an iterative algorithm that maximizes the local density by segregating the epochs based on the following characteristics: NREM sleep displayed high delta and sigma power with low EMG power, REM sleep displayed high theta power and low EMG power, and wake displayed high EMG power and low delta and sigma power. The distribution of clusters around the estimated centroid were approximated by Gaussian models assuming ellipsoidal shapes. The covariance matrix of the Gaussian models was used to calculate the Mahalanobis distance from a given epoch to each cluster. The epochs are assigned to a sleep stage according to a minimum Mahalanobis distance to the respective cluster.

All epochs identified to contain SWDs were classed separately from the other three brain states. An additional peak detection algorithm^6^ was required to identify SWDs during wake epochs. This algorithm utilises z-scores to identify peaks by calculating a moving mean and flagging datapoints that deviate from the moving mean by a given threshold. If peaks were identified in both 5–9 Hz and 10–18 Hz, this was indicative of the presence of harmonics, which are evident in SWD epochs. After automated scoring of all additional SWD epochs the sleep quantification was adjusted accordingly. SWD distribution across wake and REM was determined by evaluating the first 5 second epoch prior to a seizure occurring, while the first 30 seconds (six epochs) prior were used to assess SWD initiation in NREM and wake-NREM transitions.

After initial brain state scoring, further processing was performed on the data to remove epochs with excessive (larger than 3000 mV) noise. A third-order bandpass Butterworth filter (0.2–100 Hz) was applied to the full-length raw data. 5 second epochs were extracted from the filtered recording and discarded if any data point exceeded 3000 mV, which was beyond the range of physiological activity.

To calculate the power spectral density, brain state epoch averages were Hanning-tapered and Fourier transformed using the SciPy welch method (with 50% overlapping windows). The spectral slope, a regression-based fitting measurement which describes the rate of change of the EEG power spectra, was calculated between 1–48 Hz using the NumPy polyfit function.^7^ Epochs that were outside of the visually selected threshold of -5 log_10_(mV^2^)/Hz for the spectral slope and 500 mV for the spectral offset were removed from further analyses. Spectral power for each epoch was baseline-corrected by normalizing to the average spectral power across REM, NREM, and wake for each animal.

We validated automated sleep scoring performance by comparing the automatically generated vigilance state output with visual EEG state scoring of identical 5 second epochs. Researchers visually scoring were blind to automated output. We manually scored 4 hours of EEG in seventeen animals, between 10:00 – 14:00 o’clock, when approximately equal distributions of wake, NREM and REM epochs were observed. Percent agreement and Cohen’s kappa were calculated to assess interrater reliability between visual and automatic scoring of brain states.^8^ In our datasets, agreement between visual and automatic scoring was 88.1%, 87.5% and 95% for REM, NREM and wake states respectively. Overall agreement between the scoring methods was 90.8%. The kappa coefficient was 0.83 (± 0.002 SE) (Supplementary Fig. 1, Supplementary Table 1).

SWDs and brain state data was analysed over 48 hours starting at zeitgeber time 07:00 am of the day after connection, under a 12-hour/12-hour light–dark schedule. Values per hour over the two days were then averaged over the 24-hour period. The automated sleep scoring code is available at <https://github.com/Gonzalez-Sulser-Team/AUTOMATIC-SLEEP-SCORER>.

**Pharmacology Experiments**

Drugs were dissolved in 1 ml of sterile 0.9% saline and rats received treatment based on their weight. Each animal received either intraperitoneal ethosuximide (100 mg/kg, Merck, Germany), memantine (5 mg/kg, Enzo Life Sciences, United States), or saline as a vehicle control on individual treatment days (Fig 6A), between baseline and washout days, with 24 hours between each experimental day (baseline, drug and washout) (Fig 6A). The half-lives of ethosuximide and memantine have been reported to be 10-16 hours and less than 4 hours respectively in rats,^9,10^ suggesting that a 24-hour washout is sufficient to record a new baseline period. Treatment order (including saline) was randomized and systematically weighted so that each possible permutation of treatment order occurred across an equal number of animals to minimize the risk of synergistic effects between drugs. SWD number for each treatment per individual rat is normalized to baseline SWDs and data is presented as a percentage of the animal’s baseline SWDs. These percentages are then compared statistically across the population. Experimenters were blinded to treatment.

In a separate experiment, we infused ethosuximide and memantine into the nRT, which is thought to be involved in SWD activity in other rodent models of absence seizures and patients.^11,12^ Ethosuximide and memantine were dissolved in sterile 0.9% saline to final concentrations of 708 mM and 28 mM respectively. Treatment administration and timeline followed were as described in systemic experiments above. A total volume of 1 μl was infused for each treatment, with 0.5 μl administered per hemisphere using an infusion cannula connected by polyethylene tubing to a 1μl Hamilton syringe. Injected volume (0.5 μl) and flow rate (10 nl/s) were controlled by a precision pump (World Precision Instruments, Hertfordshire, United Kingdom). During treatment administration rats were put under mild isoflurane anaesthesia, maintained between 1.5-2.5%, and the procedure was performed under a microscope to ensure infusion was effectively achieved. Drug infusions, under anaesthesia did not exceed 10 minutes (including brief anaesthetic induction). A 1-hour period between 11:00 to 12:00 o’clock across days, was used to analyse the effects of pharmacological treatments on SWDs. Based off the pharmacokinetic two-compartment model, the half-life for isoflurane elimination from the brain of rabbits is 26 minutes.^13^ Since the rat metabolism is slightly faster than that of rabbits,^14^ and isoflurane exposure during infusions was short and at low concentrations, we reasoned that at 30 minutes most of the isoflurane is eliminated from the rat brain, while the following 1-hour period would still allow for observation of treatment effects. Treatment randomisation and SWD normalisation followed the same methods as for systemic administration and experimenters were blinded to treatment and genotype.

**Statistical Analysis**

Normality and homoscedasticity for all data were estimated by Shapiro–Wilk and Levene’s tests (rejection value set at < 0.05), and based on these, comparisons between genotypes were made using two-sample unpaired t-test, Welch’s two-sample t-test or Wilcoxon rank sum test. Two-way ANOVA tests were performed where more than two groups were compared (genotype and frequency). We use a Linear Mixed Model approach for time series analysis, where multiple measures were taken from the same subjects. Wherever appropriate, these were followed by *post hoc* Tukey’s multiple comparisons. Pearson correlation was used to test the relationship of SWDs with brain state abnormalities. Analyses were conducted in R,^5^ RStudio,^15^ using the *ggplot2*, *car* and *lmerTest* packages.^16–18^ All the data in the figures were presented as mean ± SEM, and *P* < 0.05 was considered as statistically significant and indicated with **P* < 0.05, ***P* < 0.01, ****P* < 0.001, *****P <* 0.0001.

**References**

1. Dunkley PR, Jarvie PE, Robinson PJ. A rapid Percoll gradient procedure for preparation of synaptosomes. Nat Protoc. 2008 Nov 16;3(11):1718–28.

2. Childers DG, Skinner DP, Kemerait RC. The cepstrum: A guide to processing. Proceedings of the IEEE. 1977;65(10):1428–43.

3. Madrid-López N, Estrada J, Díaz J, Bassi A, Délano PH, Ocampo-Garcés A. The Sleep–Wake Cycle in the Nicotinic Alpha-9 Acetylcholine Receptor Subunit Knock-Out Mice. Front Cell Neurosci. 2017 Oct 10;11.

4. Rahim KJ, Burr WS, Thomson DJ. Applications of Multitaper Spectral Analysis to Nonstationary Data [Internet]. Queen’s University; 2014 [cited 2024 Feb 13]. Available from: https://CRAN.R-project.org/package=multitaper

5. R Core Team. R: A Language and Environment for Statistical Computing [Internet]. Vienna, Austria; 2022. Available from: https://www.R-project.org/

6. van Brakel JPG. Robust peak detection algorithm using z-scores. 2014; Available from: https://stackoverflow.com/questions/22583391/peak-signal-detection-in-realtime-timeseries-data/22640362#22640362

7. Fasol MCM, Escudero J, Gonzalez-Sulser A. Single-Channel EEG Artifact Identification with the Spectral Slope. In: 2023 IEEE International Conference on Bioinformatics and Biomedicine (BIBM). IEEE; 2023. p. 2482–7.

8. McHugh ML. Interrater reliability: the kappa statistic. Biochem Med (Zagreb). 2012;276–82.

9. Löscher W. The Pharmacokinetics of Antiepileptic Drugs in Rats: Consequences for Maintaining Effective Drug Levels during Prolonged Drug Administration in Rat Models of Epilepsy. Epilepsia. 2007 Jul 18;48(7):1245–58.

10. Beconi MG, Howland D, Park L, Lyons K, Giuliano J, Dominguez C, et al. Pharmacokinetics of memantine in rats and mice. PLoS Curr. 2011 Dec 15;3:RRN1291.

11. Crunelli V, Lőrincz ML, McCafferty C, Lambert RC, Leresche N, Di Giovanni G, et al. Clinical and experimental insight into pathophysiology, comorbidity and therapy of absence seizures. Brain. 2020 Aug 1;143(8):2341–68.

12. Lindquist BE, Timbie C, Voskobiynyk Y, Paz JT. Thalamocortical circuits in generalized epilepsy: Pathophysiologic mechanisms and therapeutic targets. Neurobiol Dis. 2023 Jun;181:106094.

13. Wyrwicz A, Conboy C, Ryback K, Nichols B, Eisele P. In vivo 19F-NMR study of isoflurane elimination from brain. Biochimica et Biophysica Acta (BBA) - Molecular Cell Research. 1987 Jan 19;927(1):86–91.

14. Subcommittee on Laboratory Animal Nutrition, Committee on Animal Nutrition, Board on Agriculture, National Research Council, Institute for Laboratory Animal Research. Nutrient Requirements of the Laboratory Rat. In: Nutrient Requirements of Laboratory Animals. Fourth Edition. Washington DC, USA: National Academies Press; 1995.

15. Posit team. RStudio: Integrated Development Environment for R [Internet]. Boston, MA; 2022. Available from: http://www.posit.co/

16. Fox J, Weisberg S. An R Companion to Applied Regression [Internet]. Third. Thousand Oaks CA: Sage; 2019. Available from: https://socialsciences.mcmaster.ca/jfox/Books/Companion/

17. Kuznetsova A, Brockhoff PB, Christensen RHB. lmerTest Package: Tests in Linear Mixed Effects Models. J Stat Softw. 2017;82(13).

18. Wickham H. ggplot2. Cham: Springer International Publishing; 2016.
